# Versatile aliphatic polyester biosynthesis system for producing random and block copolymers composed of 2-, 3-, 4-, 5-, and 6-hydroxyalkanoates using the sequence-regulating polyhydroxyalkanoate synthase PhaC_AR_

**DOI:** 10.1186/s12934-022-01811-7

**Published:** 2022-05-14

**Authors:** Keigo Satoh, Tomoya Kawakami, Nagi Isobe, Loïc Pasquier, Hiroya Tomita, Manfred Zinn, Ken’ichiro Matsumoto

**Affiliations:** 1 Graduate School of Chemical Sciences and Engineering, Hokkaido University, N13W8, Kitaku, Sapporo, 060-8628 Japan; 2 Department of Engineering, Hokkaido University, N13W8, Kitaku, Sapporo, 060-8628 Japan; 3 Institute of Life Technologies, University of Applied Sciences and Arts Western Switzerland (HES-SO Valais-Wallis), Sion, Switzerland; 4 Division of Applied Chemistry, Faculty of Engineering, Hokkaido University, N13W8, Kitaku, Sapporo, 060-8628 Japan

**Keywords:** PHA synthase, Block copolymer, 2-Hydroxybutyrate, 4-Hydroxy-2-methylbutanoate, 5-Hydroxypentanoate, δ-Valerolactone, ε-Caprolactone, Sequence regulation, Biodegradable plastic

## Abstract

**Background:**

Polyhydroxyalkanoates (PHAs) are microbial polyesters synthesized by PHA synthases. Naturally occurring PHA copolymers possess a random monomer sequence. The development of PhaC_AR_, a unique sequence-regulating PHA synthase, has enabled the spontaneous biosynthesis of PHA block copolymers. PhaC_AR_ synthesizes both a block copolymer poly(2-hydroxybutyrate)-*b*-poly(3-hydroxybutyrate) [P(2HB)-*b*-P(3HB)], and a random copolymer, poly(3HB-*co*-3-hydroxyhexanoate), indicating that the combination of monomers determines the monomer sequence. Therefore, in this study, we explored the substrate scope of PhaC_AR_ and the monomer sequences of the resulting copolymers to identify the determinants of the monomer sequence. PhaC_AR_ is a class I PHA synthase that is thought to incorporate long-main-chain hydroxyalkanoates (LMC HAs, > C_3_ in the main [backbone] chain). Thus, the LMC monomers, 4-hydroxy-2-methylbutyrate (4H2MB), 5-hydroxyvalerate (5HV), and 6-hydroxyhexanoate (6HHx), as well as 2HB, 3HB, and 3-hydroxypropionate (3HP) were tested.

**Results:**

Recombinant *Escherichia coli* harboring PhaC_AR_, CoA transferase and CoA ligase genes was used for PHA production. The medium contained the monomer precursors, 2HB, 3HB, 3HP, 4H2MB, 5HV, and 6HHx, either individually or in combination. As a result, homopolymers were obtained only for 3HB and 3HP. Moreover, 3HB and 3HP were randomly copolymerized by PhaC_AR_. 3HB-based binary copolymers P(3HB-*co*-LMC HA)s containing up to 2.9 mol% 4H2MB, 4.8 mol% 5HV, or 1.8 mol% 6HHx were produced. Differential scanning calorimetry analysis of the copolymers indicated that P(3HB-*co*-LMC HA)s had a random sequence. In contrast, combining 3HP and 2HB induced the synthesis of P(3HP)-*b*-P(2HB). Similarly, P(2HB) segment-containing block copolymers P(3HB-*co*-LMC HA)-*b*-P(2HB)s were synthesized. Binary copolymers of LMC HAs and 2HB were not obtained, indicating that the 3HB or 3HP unit is essential to the polymer synthesis.

**Conclusion:**

PhaC_AR_ possesses a wide substrate scope towards 2-, 3-, 4-, 5-, and 6-hydroxyalkanoates. 3HB or 3HP units are essential for polymer synthesis using PhaC_AR_. The presence of a 2HB monomer is key to synthesizing block copolymers, such as P(3HP)-*b*-P(2HB) and P(3HB-*co*-LMC HA)-*b*-P(2HB)s. The copolymers that did not contain 2HB units had a random sequence. This study’s results provide insights into the mechanism of sequence regulation by PhaC_AR_ and pave the way for designing PHA block copolymers.

**Supplementary Information:**

The online version contains supplementary material available at 10.1186/s12934-022-01811-7.

## Background

Aliphatic polyesters comprising hydroxycarboxylates, such as microbial polyhydroxyalkanoate (PHA), chemically synthesized polylactic acid (PLA), and petrol-based polycaprolactone (PCL), are attracting considerable research interest due to their applicative physical properties and degradability in natural environments [1, 2], compost or both [3, 4]. PCL and PLA are typically synthesized via ring-opening polymerization (ROP) of corresponding lactones using inorganic, organic, or enzymatic catalysts [5–7]. In contrast, PHAs are produced in microbial cells from hydroxyacyl-coenzyme A (HA-CoA)s via successive transesterification by PHA synthase activity [8]. PHA biosynthesis systems have several advantages over ROP. PHAs are produced via a one-step fermentation process from different feedstock [9, 10]. PHA synthases possess strict enantio-specificity and synthesize isotactic polymers [11]. For certain types of polymers, PHA synthases synthesize polymers with a relatively high molecular weight [12] compared to those synthesized by ROP. Thus, PHA synthase plays a critical role in PHA biosynthesis. In addition, PHAs are well biodegraded in various environments, partly because natural polymers induce the expression of degrading enzymes by microorganisms [13].

Naturally occurring PHAs comprise (*R*)-3-hydroxyalkanoates (3HAs) with 4–12 carbons [11]. Among these, poly(3-hydroxybutyrate) [P(3HB)] is the most abundant PHA in nature and is produced by many microbes. Although the brittleness of P(3HB) has limited its practical use, recent studies have developed several applications by blending P(3HB) with other polymers, such as soy protein fibers [14], chitosans [15], and PLAs [16]. Another effective strategy to reduce brittleness is random copolymerization, which induces increased ductility of the polymer. A random copolymer, P(3-hydroxybutyrate-*co*-3-hydroxyhexanoate) [P(3HB-*co*-3HHx) or PHBHx], has been extensively studied [17] and commercially manufactured and implemented as a commodity plastic [18]. Natural PHA copolymers possess a random sequence [19] presumably because the monomer supply and polymerization proceed simultaneously.

The limitation of the structural variety of PHA has been recognized as a drawback in practical applications of the material. Therefore, exploring PHA synthases with a wide substrate scope—in other words, low substrate specificity—has been an important research target [20, 21]. In addition, enzyme engineering is an effective approach for further expanding its substrate scope [22]. In particular, the discovery of 2-hydroxyalkanoate (2HA)-incorporating PHA synthase significantly expanded the structural variety of PHAs. A pairwise mutant of class II PHA synthase from *Pseudomonas* sp. 61–3 (PhaC1_Ps_STQK) was the first enzyme that incorporated 2HAs into the polymer [23] (for details of PHA synthase classification, see [8, 24, 25]). PhaC1_Ps_STQK incorporates lactate (LA, 2-hydroxypropionate) [23], glycolate (GL, 2-hydroxyacetate) [26], 2-hydroxybutyrate (2HB) [27, 28], and amino acid-derived 2HAs [29]. A random copolymer of LA and medium-chain-length (MCL) 3HA-CoAs (C_6–12_) was also synthesized using the same enzyme [30]. Strict enantio-specificity of PHA synthase has enabled the synthesis of highly isotactic polymers from inexpensive racemic precursors, which is an advantage over ROP. This has also enabled the characterization of the mechanical properties of P[(*R*)-2HB] [27].

The monomer sequence of copolymer is an effective factor that influences the physical properties of polymer materials. Block copolymers exhibit useful and characteristic properties, and many studies on chemical block copolymer synthesis exist [31]. Block copolymers comprising immiscible segments spontaneously form phase-separated structure on the nanoscale, known as microphase separation, that is the principle of their physical properties. On the other hand, random copolymerization is effective to reduce the crystallinity of the polymers, which typically softens the materials. As mentioned above, PHA copolymers generally possess a random sequence. Therefore, several attempts have been made to synthesize PHA block copolymers by manipulating monomer supplies during cultivation [32]. However, the difference in time required to synthesize one polymer chain and cultivation complicates this strategy [33].

We previously reported that PhaC_AR_—a chimeric class I PHA synthase comprising PhaCs from *Aeromonas caviae* and *Ralstonia eutropha* (formally *Cupriavidus necator*) [34]—has a unique sequence-regulating capacity and spontaneously synthesizes block copolymers from a mixture of substrates [33]. In addition, PhaC_AR_ can efficiently incorporate 2HB and GL units into polymers. Using this enzyme, PHA block copolymers, P(2HB)-*b*-(3HB) [33], P(GL-*ran*-3HB)-*b*-P(3HB) [35], and P(GL-*co*-3HB-*co*-3HHx)-*b*-P(3HB-*co*-3HHx) [36] were synthesized. P(2HB)-*b*-(3HB) exhibits elastomer-like properties that characterize block copolymers [37]. These results also demonstrate that PhaC_AR_ synthesizes block and random copolymers, depending on the monomer combination. Nevertheless, the regularity of monomer sequence formation by PhaC_AR_, i.e., which monomers trigger random or block sequences, remains unsolved.

As aforementioned, PhaC_AR_ is a class I 2HA-incorporating PHA synthase. Class I PHA synthases can recognize unusual long-main-chain hydroxyalkanoates (LMC HAs, ≥ C_4_ in the main-chain) monomers, such as 4-hydroxybutyrate (4HB) and 5-hydroxyvalerate (5HV). 4HB-containing PHAs have been extensively studied and used in biomedical applications (for details, see review [38]). The inefficiency of P(4HB) synthesis by ROP makes the biosynthetic process attractive [39]. P[3HB-*co*-3-hydroxypropionate (3HP)-*co*-5HV] was produced using *R. eutropha*, which possesses the class I PHA synthase PhaC_Re_ [40]. Little is known about 6HHx-containing PHAs. P(3HB-*co*-4HB-*co*-6HHx) was synthesized by *Methylocystis parvus*, which has uncharacterized PHA synthase [41]. The 3HP and 4HB units in these polymers were generated via β-oxidation of 5HV and 6HHx, respectively. Meanwhile, a binary copolymer, P(3HB-*co*-6HHx), was chemically synthesized by ROP of (*R*)-β-butyrolactone (92% ee) and ε-caprolactone [42]. The copolymer had a lower glass transition temperature (*T*_g_), which is approaching − 67 °C depending on its 6HHx fraction, than homopolymeric P(3HB) (4 °C). Similarly, P(3HB-*co*-5HV) was synthesized by ROP of (*R*)-β-butyrolactone and δ-valerolactone [43].

3HP possesses the same main-chain structure as 3HB and shares structural characteristics with the LMC monomers regarding the terminal hydroxy group. 3HP units are incorporated by some class I enzymes [44]. P(3HP) and its copolymers have not been found in nature, but they can be synthesized by natural class I PHA synthases. Glycerol [45], β-alanine [46], and exogenously supplemented 3HP are used as precursors of 3HP units. The use of nontoxic starting substances is a benefit of P(3HP) biosynthesis compared to ROP of β-propiolactone [47] with a carcinogenic effect [48]. P(3HP) has high tensile strength and stretchable properties, and it is degraded by PHA depolymerase activity [44, 49].

These studies on class I PHA synthases combined with our previous results of PhaC_AR_ suggest that PhaC_AR_ potentially possesses a wide substrate scope toward 3HB and 2HB as well as 3HP and LMC monomers. In addition, the monomer sequence of the obtained polymers is imperative. Here we attempted to synthesize homopolymers and copolymers containing 3HB, 3HP, 2HB, and LMC monomers and analyzed their monomer sequences. 4-Hydroxy-2-methylbutyrate (4H2MB) was used as a 4HB analogue. The experiments also aimed at identifying the determinants of the monomer sequence of copolymers synthesized using PhaC_AR_. Consequently, we demonstrated that PhaC_AR_ provides a versatile system for producing various random and block aliphatic copolyesters.

## Results

### Preparation of LMC HA monomer precursors

**Fig. 1:**
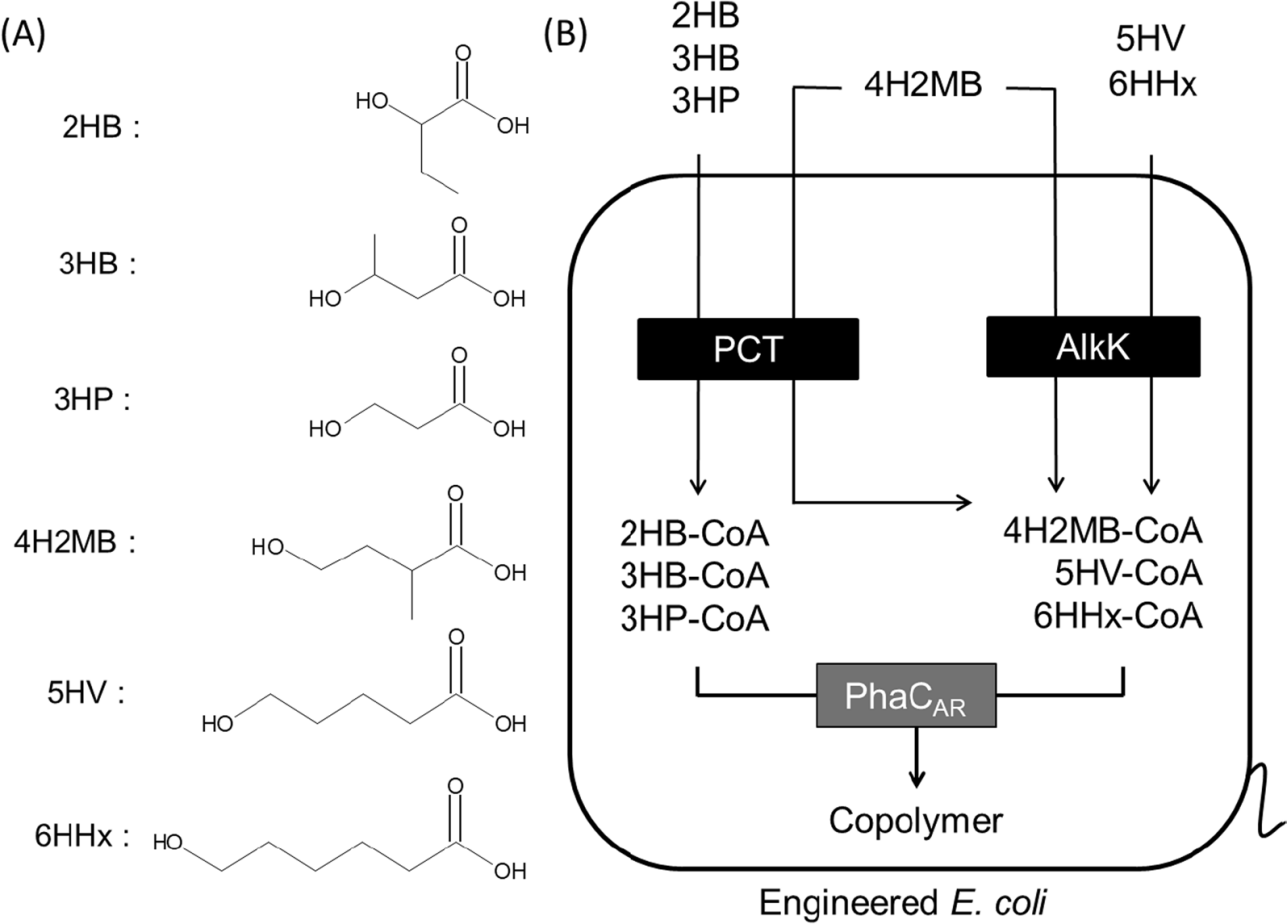
**A** Structure of the monomers used in the PHA synthesis in this study and **B** metabolic pathways for the production of sequence-regulated PHAs containing long-main-chain monomers. 2HB, 3HB, and 3HP are converted to the corresponding CoA thioesters by PCT. 4H2MB is converted by PCT and AlkK. 5HV and 6HHx are converted by AlkK. These CoA thioesters obtained here are used as the substrates for the polymerase reaction

Monomer precursors, 4H2MB, 5HV, and 6HHx (Fig. 1A), were prepared by hydrolyzing their corresponding lactones. To avoid unintended ROP, the lactones were hydrolyzed in a mild condition as described in the Methods section. After hydrolysis, the reaction mixtures contained no polymerized products based on diffusion ordered spectroscopy (DOSY) nuclear magnetic resonance (NMR) analysis (Additional file 1: Fig. S1A–C).

### 3HB-based binary copolymer synthesis

The synthesis of 3HB-based binary copolymers containing 3HP, 4H2MB, 5HV, and 6HHx as secondary monomer units was attempted. It is known that propionyl-CoA transferase (PCT) from *Methanosphaera elsdenii* can convert short-chain-length (SCL, ≤ C_5_) 2HAs and 3HAs including 2HB and 3HB to 2HA/3HA-CoAs using acetyl-CoA as the CoA donor [27]. CoA ligase AlkK catalyzes the condensation of MCL 3HA and CoA using ATP [50][51]. In this study, these enzymes were used to supply 3HP-CoA and LMC HA-CoAs from the corresponding precursors. The polymer production in the presence (**Entry 5–8**) and absence (**Entry 1–4**) of AlkK was compared to estimate the substrate specificity of AlkK toward these substrates. Consequently, the copolymer production was observed under all conditions (Table 1, **Entry 1–8**). The 4H2MB fraction with AlkK exceeded that without AlkK; 5HV and 6HHx units were incorporated with AlkK but not with PCT alone. These results indicate that AlkK is effective for supplying the LMC monomers, and PCT has no activity toward 5HV and 6HHx. In addition, PhaC_AR_ was found to polymerize 3HP-CoA and LMC-HA-CoAs as substrates. Figure 1B shows the proposed pathway based on the results.

**Table 1:** Production of 3HB-based copolymers and various homopolymers with and without AlkK

Monomer supplying genes ^a^	Entry	Precursor concentration (g L^−1^) ^b^		Cell dry weight (g L^−1^)	Polymer production (g L^1^)	Monomer composition (mol%)						*M*_w_ (× 10^5^)	*M*_n_ (× 10^5^)	*M*_w_/*M*_n_
						2HB	3HB	3HP	4H2MB	5HV	6HHx			
*pct*	1	3HB 2.5	3HP 1.0	3.67 ± 0.07	0.49 ± 0.10	–	51.4	48.6	–	–	–	6.3	2.8	2.3
*pct*	2	3HB 2.5	4H2MB 1.0	3.68 ± 0.06	0.48 ± 0.03	–	97.9	–	2.1	–	–	2.9	0.8	3.6
*pct*	3	3HB 2.5	5HV 1.0	3.17 ± 0.05	0.32 ± 0.02	–	100	–	–	–	–	2.0	0.9	2.3
*pct*	4	3HB 2.5	6HHx 1.0	3.10 ± 0.04	0.33 ± 0.03	–	100	–	–	–	–	1.7	0.7	2.5
*pct*, *alkK*	5	3HB 2.5	3HP 1.0	3.70 ± 0.13	0.78 ± 0.13	–	55.5	44.5	–	–	–	4.6	2.3	2.0
*pct*, *alkK*	6	3HB 2.5	4H2MB 1.0	3.32 ± 0.21	0.66 ± 0.03	–	97.1	–	2.9	–	–	1.9	0.9	2.1
*pct*, *alkK*	7	3HB 2.5	5HV 1.0	2.81 ± 0.38	0.42 ± 0.06	–	95.3	–	–	4.7	–	1.1	0.5	2.2
*pct*, *alkK*	8	3HB 2.5	6HHx 1.0	2.85 ± 0.08	0.34 ± 0.02	–	98.2	–	–	–	1.8	0.8	0.4	2.1
*pct*, *alkK*	9	–	2HB 2.5	2.61 ± 0.10	ND^c^	–	–	–	–	–	–	–	–	–
*pct*, *alkK*	10	–	3HB 2.5	3.61 ± 0.07	0.44 ± 0.05	–	100	–	–	–	–	2.0	1.1	1.8
*pct*, *alkK*	11	–	3HP 1.0	3.03 ± 0.08	0.25 ± 0.02	–	–	100	–	–	–	3.2	1.7	1.8
*pct*, *alkK*	12	–	4H2MB 1.0	3.37 ± 0.63	ND	–	–	–	–	–	–	–	–	–
*pct*, *alkK*	13	–	5HV 1.0	2.62 ± 0.10	ND	–	–	–	–	–	–	–	–	–
*pct*, *alkK*	14	–	6HHx 1.0	2.44 ± 0.09	ND	–	–	–	–	–	–	–	–	–

^a^ pBSP_Re_phaC_AR_pct and pBSP_Re_phaC_AR_pctalkK were used respectively. ^b^ The concentrations of precursors are given as the sodium salts. ^c^ ND, not detected

The polymer samples (**Entry 5–8**) were subjected to gas chromatography (GC) analysis to further verify the incorporation of 3HP and LMC HA units. From Additional file 1: Fig. S2A–N, the methanolysis products of the polymers were identical to those from monomer standards. The detected components were consistent with the NMR results. The results indicate that 3HP and LMC HA units were incorporated into the copolymers.

Next, homopolymer production of 2HB, 3HB, 3HP, and LMC HAs was attempted. Polymer production was observed only for 3HB and 3HP (Table 1, **Entry 9–14**).

### 3HP-based binary copolymer synthesis

Because PhaC_AR_ synthesized P(3HP), we subsequently attempted 3HP-based binary copolymer production containing LMC HAs as the secondary monomer units. However, no LMC HAs were incorporated and P(3HP) was obtained under these conditions (Table 2, **Entry 15–17**). Similarly, no polymer was obtained in the combination of 2HB and LMC HAs (Table 2, **entry 20–22**). In contrast, synergizing 3HP and 2HB induced copolymer production. Overall, it was found that 3HB or 3HP is essential for polymerization using PhaC_AR_ among the tested monomers, and that LMC HAs were incorporated only in the presence of 3HB.

**Table 2:** Production of 3HP- and 2HB-based polymers

Monomer supplying genes	Entry	Precursor concentration (g L^−1^)^a^		Cell dry weight (g L^−1^)	Polymer production (g L-^1^)	Monomer composition (mol%)						*M*_w_ (× 10^5^)	*M*_n_ (× 10^5^)	*M*_w_/*M*_n_
						2HB	3HB	3HP	4H2MB	5HV	6HHx			
*pct, alkK*	15	3HP 1.0	4H2MB 1.0	3.53 ± 0.23	0.18 ± 0.05	–	–	100	–	–	–	2.2	1.0	2.3
*pct, alkK*	16	3HP 1.0	5HV 1.0	3.20 ± 0.07	0.19 ± 0.04	–	–	100	–	–	–	3.4	1.4	2.4
*pct, alkK*	17	3HP 1.0	6HHx 1.0	3.06 ± 0.06	0.19 ± 0.01	–	–	100	–	–	–	2.7	1.2	1.6
*pct, alkK*	18	2HB 2.5	3HB 2.5	3.18 ± 0.05	0.40 ± 0.04	32.5	67.5	-	–	–	–	2.8	0.9	3.0
*pct, alkK*	19	2HB 2.5	3HP 1.0	2.61 ± 0.13	0.16 ± 0.02	4.2	-	95.8	–	–	–	1.9	1.2	1.6
*pct, alkK*	20	2HB 2.5	4H2MB 1.0	2.46 ± 0.24	ND	–	–	–	–	–	–	–	–	–
*pct, alkK*	21	2HB 2.5	5HV 1.0	2.31 ± 0.20	ND	–	–	–	–	–	–	–	–	–
*pct, alkK*	22	2HB 2.5	6HHx 1.0	2.47 ± 0.03	ND	–	–	–	–	–	–	–	–	–

^a^ The concentrations of precursors are given as the sodium salts

### Synthesis of 2HB-containing terpolymers

2HB-containing ternary copolymer production was conducted using 3HB, 3HP, and LMC HAs in combination. Consequently, ternary copolymers were produced under all conditions (Table 3). The 3HP fraction in P(2HB-*co*-3HP-*co*-3HB) significantly exceeded those of 4H2MB, 5HV, and 6HHx fractions in their corresponding copolymers, indicating the preference of PhaC_AR_ for 3HB and 3HP monomers.

**Table 3:** Biosynthesis of 2HB-containing ternary copolymers

Monomer supplying genes	Entry	Precursor concentration (g L^−1^)^a^			Cell dry weight (g L^−1^)	Polymer production (g L^−1^)	Monomer composition (mol%)						*M*_w_ (× 10^5^)	*M*_n_ (× 10^5^)	*M*_w_/*M*_n_
							2HB	3HB	3HP	4H2MB	5HV	6HHx			
*pct, alkK*	23	2HB 2.5	3HP 1.0	3HB 2.5	3.10 ± 0.03	0.26 ± 0.03	4.4	57.2	38.3	–	–	–	3.7	1.2	3.2
*pct, alkK*	24	2HB 2.5	4H2MB 1.0	3HB 2.5	3.53 ± 0.16	0.39 ± 0.03	26.8	71	–	2.2	–	–	2.3	1.0	2.4
*pct, alkK*	25	2HB 2.5	5HV 1.0	3HB 2.5	3.15 ± 0.07	0.45 ± 0.03	31.6	65.3	–	–	3.1	–	1.6	0.7	2.4
*pct, alkK*	26	2HB 2.5	6HHx 1.0	3HB 2.5	2.98 ± 0.06	0.33 ± 0.01	25.3	73.6	–	–	–	1.1	1.1	0.5	1.9

^a^ The concentrations of precursors are shown as the sodium salts

### Sequence analysis of 3HB-based binary copolymers

The thermal properties of P(3HB-*co*-LMC HAs) were analyzed by differential scanning calorimetry (DSC) (Table 4) to determine their monomer sequences. The melting point of P(3HB) homopolymer exceed those of P(3HB-*co*-4H2MB), P(3HB-*co*-5HV), and P(3HB-*co*-6HHx), indicating that 4H2MB, 5HV, and 6HHx were randomly introduced into the polymer chains, and they reduced the crystallinity of P(3HB). The 3HB and 3HP units were biasedly copolymerized by ^13^C NMR analysis (Additional file 1: Fig. S3), as the dyads of 3HB-3HB and 3HP-3HP were more abundant than those of 3HB-3HP and 3HP-3HB. In addition, the copolymer exhibited two melting points. These results suggest that P(3HB-*co*-3HP) synthesized by PhaC_AR_ is a random copolymer, which possesses 3HB-rich and 3HP-rich heterogeneous copolymer fractions.

**Table 4:** DSC analysis of homopolymers and copolymers synthesized by PhaC_AR_

Sample	Synthesis conditions^a^	*T*_g_ (°C)	*T*_*m*_ (°C)	ΔH (J/g)
P(3HP)	Entry 11	− 15.0	71.9	62.3
P(3HB)	Entry 10	2.4	169.0	65.9
P(55.5 mol% 3HB-*co*-3HP)	Entry 1	− 15.5, 0.8	47.9, 161.1	7.7, 10.0
P(97.1 mol% 3HB-*co*-4H2MB)	Entry 6	1.1	155.9	55.5
P(95.2 mol% 3HB-*co*-5HV)	Entry 7	− 2.1	159.2	49.4
P(98.3 mol% 3HB-*co*-6HHx)	Entry 8	0.0	162.4	59.0
P(28.9 mol% 2HB-*co*-3HB)	Entry 18	3.0	163.4	28.8
P(4.8 mol% 2HB-*co*-3HP)	Entry 19	− 14.6	73.2	55.1
P(4.4 mol% 2HB-*co*-3HB-*co*-3HP 55.1 mol%)	Entry 23	− 11.8, − 1.3	50.3	2.3
P(26.8 mol% 2HB-*co*-3HB-*co*-4H2MB 2.2 mol%)	Entry 24	2.7	159.3	35.9
P(32.4 mol% 2HB-*co*-3HB-*co*-5HV 3.8 mol%)	Entry 25	2.0	163.7	30.3
P(26.5 mol% 2HB-*co*-3HB-*co*-6HHx 1.1 mol%)	Entry 26	1.7	159.4	27.6

^a^ The polymers were synthesized using conditions in Tables 1, 2 and 4. Combining different batches caused the slight difference in the monomer composition. Thermograms are shown in Additional file 1: Fig. S5AB

### Sequence analysis of 2HB-containing copolymers

**Fig. 2:**
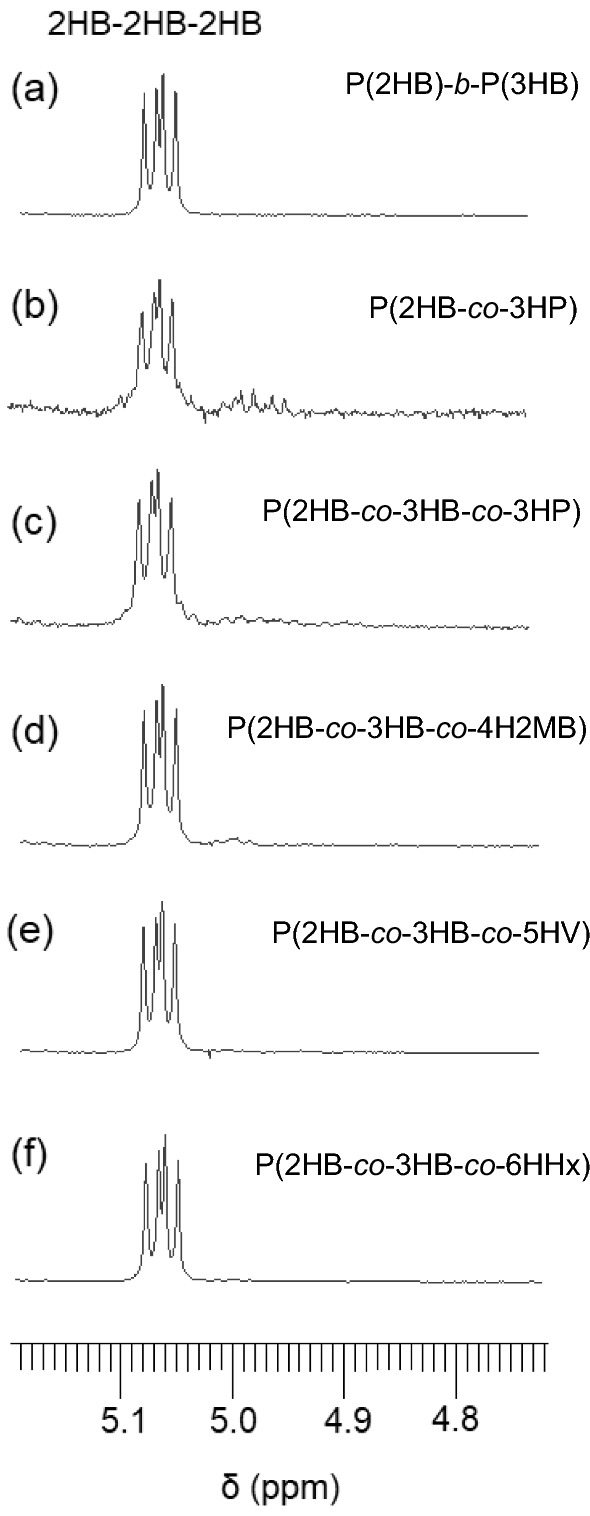
^1^H NMR analysis of 2HB-containing polymers. The resonance of methine proton of 2HB units is magnified

The monomer sequence of the 2HB-containing segments is estimated based on triad sequence of the 2HB units, which is determined using ^1^H NMR [37]. Figure 2 shows the magnified ^1^H NMR resonance ascribed to the methine proton of 2HB units (full spectra are shown in Additional file 1: Fig S4). All polymers exhibited the same resonance corresponding to the 2HB-2HB*-2HB triad. The results suggest that the 2HB-containing copolymers have a P(2HB) homopolymer segment. DSC analysis did not detect a melting peak of P(2HB) crystals in 2HB-containing copolymers (Table 4), probably due to the low 2HB fraction and slow crystallization of P(2HB).

**Fig. 3:**
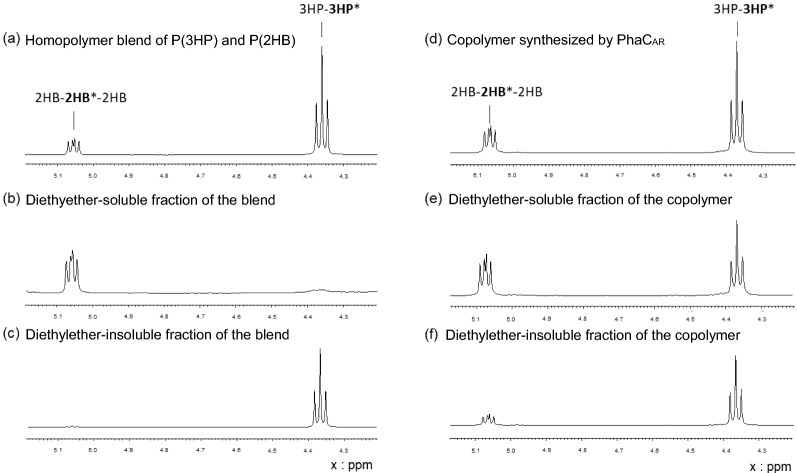
^1^H NMR of diethylether-soluble and insoluble fractions of P(3HP-*co*-2HB) synthesized using PhaC_AR_ and homopolymer blend

NMR analysis clearly distinguishes random and block copolymers, but it detects no difference between a block copolymer and homopolymer blend. Therefore, solvent fractionation of P(3HP-*co*-2HB) was conducted to verify whether there are covalent linkages between P(3HP) and P(2HB) segments in the polymer. Diethylether dissolves P(2HB) but not P(3HP). Thus, the polymer samples were fractionated into diethylether-soluble and insoluble fractions (Table 5). This procedure completely separates a blend of two homopolymers P(2HB) and P(3HP) (Fig. 3). In contrast, for P(2HB-*co*-3HP), both signals of 2HB and 3HP were observed in both the diethylether-soluble and insoluble fractions. These results strongly indicate a covalent linkage between the P(2HB) and P(3HP) segments, thereby indicating that the copolymer of 3HP and 2HB synthesized using PhaC_AR_ is a block copolymer P(3HP)-*b*-P(2HB).

**Table 5:** Solvent fractionation of P(2HB-*co*-3HP) synthesized using PhaC_AR_

Polymers	Monomer composition (mol%)		Recovery
	3HP	2HB	(mol%)
Blend of P(3HP) and P(2HB)	65	35	100
Soluble fraction	94	6	46
Insoluble fraction	0	100	53
Original Copolymer	56	44	100
Soluble fraction	64	36	39
Insoluble fraction	39	61	52

## Discussion

**Fig. 4:**
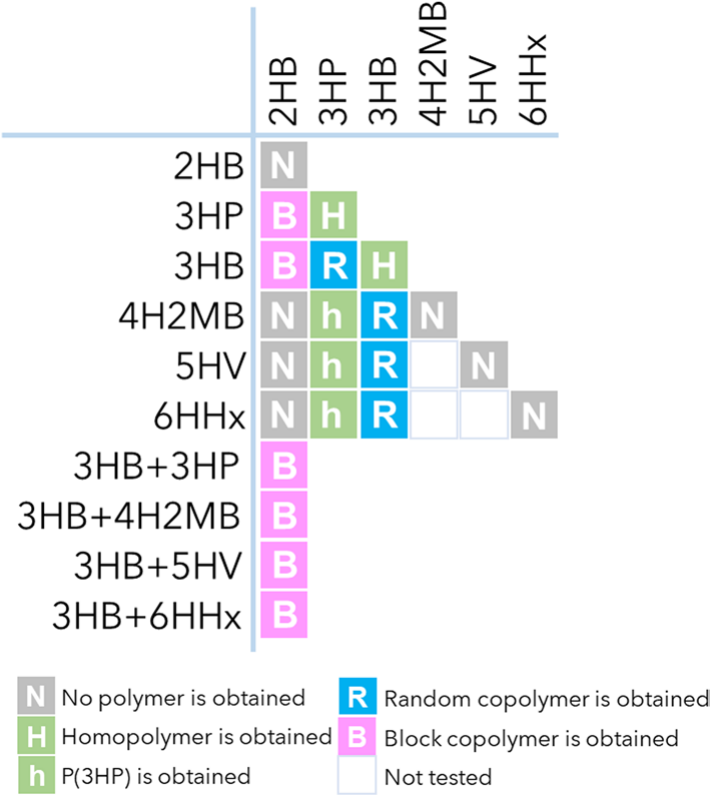
Regularity of monomer sequence by the combination of monomers. The table indicates the combinations of monomer precursors. In ternary copolymer producing conditions, the 3HB-based segment has a random sequence

In this study, we described copolymer synthesis containing 3HP, 4H2MB, 5HV, and 6HHx units that possess a terminal hydroxy group using PhaC_AR_ (Fig. 4). Together with the natural monomer unit 3HB, the four unusual monomers were successfully incorporated into their respective polymers. To our best knowledge, only one study has reported the biosynthesis of 6HHx-containing PHA [41]. For the incorporation of 2HA units, PhaC_AR_ is a unique class I PHA synthase that efficiently polymerizes 2HB-CoA. For other class I enzymes, PhaC_Re_ reportedly incorporates a small amount of 2HB units in vitro [52]. Some engineered class II enzymes, such as PhaC1_Ps_STQK, efficiently incorporate 2HB units, but they have not been reported to incorporate 3HP and LMC HAs. Therefore, PhaC_AR_ possesses the widest substrate scope for the length of the main-chain among the previously characterized PhaCs.

The LMC HA units were randomly incorporated into the P(3HB) backbone by PhaC_AR_. Random copolymerization is an effective method for reducing the crystallinity and glass transition temperature of polymers (Table 4), which contribute to improving impact resistance [42]. Similar effects can be obtained by introducing the MCL 3HA units [53, 54]. The difference between the LCM HA and MCL 3HA units is their enzymatic degradability. The presence of 4HB, 5HV, and 6HHx in PHAs enhances PHA degradability by lipases [55, 56], which could influence biodegradability in the environment. In fact, in compositing conditions, PCL [e.g. P(6HHx)] is degraded more rapidly than MCL PHA [57]. In addition, PCL and P(3HB) are marine degradable [58]. These results suggest that LCM HA units are potentially used to modulate the biodegradability of PHA and expand the type of its degrading enzymes.

A characteristic of PhaC_AR_ is its limited homopolymer-synthesizing capacity despite its wide substrate scope. Only P(3HB) and P(3HP) were obtained under single precursor supplemented conditions (Table 1). The limited capacity of homopolymer synthesis is presumably due to a potential barrier for initiating polymerization. Notably, some PHA synthases exhibit a slow reaction rate at the initial reaction stage, termed a lag phase [59]. Polymerization is accelerated in the presence of preferred substrates, which eliminates the lag phase. This study’s results indicate that 3HB-CoA and 3HP-CoA can proceed the initiation step of the polymerization while LMC HA-CoAs do not. The mature form of PhaC_AR_ is thought to accept a wider range of substrates. The requirement in the initiation would not be a critical drawback in the molecular design of block PHAs, because enzyme engineering enables the acquisition of homopolymer-synthesizing capacity [60]. We recently reported that a PhaC_AR_ derivative with reinforced activity toward 3HHx-CoA can synthesize a P(3HHx) homopolymer [61]. Given these relevant achievements, the enzymatic characteristics of PhaC_AR_ analyzed in this study provide direction(s) of its engineering for producing diverse PHA block copolymers.

Sequence regulation is a fascinating property of PhaC_AR_. In this study, a block sequence was generated when the medium was supplemented with 2HB (Figs. 2 and 4). Previous studies have shown that homo-random block sequences are formed in the presence of GL units [35]. Collectively, 2-hydroxyacyl (2HA)-CoAs are thought to trigger block copolymerization. However, the role of 2HA-CoAs in block copolymer synthesis is yet to be fully understood at the molecular level. Further studies are needed to clarify this problem.

## Conclusion

Unique sequence-regulating PHA synthase PhaC_AR_ can incorporate various HA units in the main-chain-length range of C_2–6_. The substrate scope is the widest among characterized PHA synthases. Monomers play different roles in PHA synthesis depending on their main-chain-length. 3HA units (3HB or 3HP) are essential for the polymer synthesis by PhaC_AR_ and allow the incorporation of 2HB and LMC HA units as secondary components. The presence of a 2HB monomer presumably triggers block copolymerization, and 2HB units are incorporated into the polymer chain as a P(2HB) homopolymer segment. LMC HAs are incorporated into the P(3HB) backbone with a random sequence; thus, they are effective in controlling the crystallinity of the 3HB-based segment. These findings of regularity of the sequence control are useful for the molecular design of PHA block copolymers. Finally, PhaC_AR_ provides a versatile biosynthetic system for random and block copolyesters comprising 2-, 3-, 4-, 5-, and 6-hydroxyalkanoates.

## Methods

### Bacterial strain and plasmids

*E. coli* JM109 was used as the host strain (Table 6). The plasmids pBSP_Re_phaC_AR_pct and pBSP_Re_phaC_AR_pctalkK [36] were used for polymer production. These plasmids are the pBluescript KS^+^ derivatives with the ampicillin resistance gene. pBSP_Re_phaC_AR_pct contains the *phaC*_*AR*_ gene encoding the engineered chimeric PHA synthase, the *pct* gene encoding propionyl-CoA transferase from *Megasphaera elsdenii* under the P_Re_ promoter from the *phb* operon in *R. eutropha* [33]. In addition, pBSP_Re_phaC_AR_pctalkK harbors the *alkK* gene encoding MCL 3-hydroxyalkanoic acid CoA ligase from *Pseudomonas putida* [51].

**Table 6:** Stain and plasmids used in this study

	Description	Source
Strain		
* Escherichia coli* JM109	*recA1*, *endA1*, *gyrA96*, *thi-1*, *hsdR17* (*r*_*K*_^*–*^* m*_*K*_^+^), *e14*^–^(*mcrA*^–^), *supE44*, *relA1*, Δ(*lac–proAB*)/F′ [*traD36*, *proAB*^+^, *lacI*^*q*^, *lacZ*ΔM15]	Toyobo
Plasmid		
pBSP_Re_phaC_AR_pct	pBluescript KS^+^ derivative containing the engineered chimeric PHA synthase gene *phaC*_*AR*_ and *pct* from *Megasphaera elsdenii* under the P_Re_ promoter from the *phb* operon in *Ralstonia eutropha* (*Cupriavidus necator*)	[33]
pBSP_Re_phaC_AR_pctalkK	pBSP_Re_phaC_AR_pct derivative containing *alkK* from *Pseudomonas putida*	[36]

### Culture conditions for polymer production

*E. coli* JM109 harboring pBSP_Re_phaC_AR_pctalkK was cultivated in a 1.5 mL Luria–Bertani (LB) medium (10 g/L NaCl, 10 g/L tryptone, and 5 g/L yeast extract) containing ampicillin (100 mg/L) at 30 °C overnight as a preculture. The preculture (1 mL) was used to inoculate the LB medium (100 mL) containing glucose (20 g/L), ampicillin, and monomer precursors (see below) in a 500 mL flask. Cells were cultivated with reciprocal shaking (120 rpm) at 30 °C for 48 h, then collected by centrifugation (5000×*g*, 10 min, 4 °C), washed twice with pure water, and lyophilized.

Sodium (*R,S*)-3-hydroxybutyrate (3HB-Na), sodium (*R,S*)-2-hydroxybutyrate (2HB-Na), and sodium 3-hydroxypropionate (3HP-Na) were purchased from Tokyo Chemical Industry Co., Ltd. (TCI; Tokyo, Japan). Sodium 4-hydroxy-2-methylbutyrate (4H2MB-Na), sodium 5-hydroxyvalerate (5HV-Na), and sodium 6-hydroxyhexanoate (6HHx-Na) were prepared by hydrolyzing their corresponding lactones: α-methyl-ε-butyrolactone, δ-valerolactone, and ε-caprolactone (TCI). The lactones (5 g) were added to 100 ml of 1 N NaOH and hydrolyzed at 65 °C for 3 days. The solutions were neutralized by 6 N HCl to pH 7. The absence of polymerized products in the hydrolysates was confirmed by DOSY-NMR (Additional file 1: Fig. S1A–C).

### Analysis of monomer composition

The polymers (2–5 mg/mL for ^1^H NMR, 20 mg/mL for ^13^C NMR) were placed in screwed test tubes (round bottom), dissolved in 1 mL of CDCl_3_, and heated at 60 °C for 15 min. The mixtures were then cooled to room temperature and filtered through a 0.2-μm pore size polytetrafluoroethylene (PTFE) filter. NMR spectra were obtained using a JEOL ECS-400 spectrometer. The monomer composition of the synthesized polymer was determined by ^1^H NMR. The 400 MHz ^1^H NMR spectra were recorded in CDCl_3_: P(3HB), δ 1.27 (d, 3H), 2.54 (dq, 2H), 5.26 (m, 1H). P(3HP), δ 2.67 (t, 2H), 4.37 (t, 2H). P(3HB-*co*-3HP), δ 1.28 (d, 3H), 2.44–2.64 (m, 2H), 2.67 (t, 2H), 4.37 (t, 2H), 5.26 (m, 1H). P(3HB-*co*-4H2MB), δ 1.17 (d, 3H), 1.28 (d, 3H), 2.00 (t, 1H), 2.54 (dq, 2H), 4.08–4.16 (m, 2H), 5.26 (m, 1H). P(3HB-*co*-5HV), δ 1.27 (d, 3H), 1.66 (t, 2H), 2.29–2.35 (m, 2H), 2.54 (dq, 2H), 5.26 (m, 1H). P(3HB-*co*-6HHx), δ 1.27 (d, 3H), 1.64 (t, 2H), 1.97–2.04 (m, 2H), 2.54 (dq, 2H), 4.06 (t, 2H), 5.26 (m, 1H). P(2HB-*co*-3HP), δ 1.05 (t, 3H), 1.94–2.02 (m, 2H), 2.67 (t, 2H), 4.37 (t, 3H), 5.02–5.08 (m, 1H). P(2HB-*co*-3HB), δ 1.04 (t, 3H), 1.27 (d, 3H), 1.89–2.08 (m, 2H), 2.54 (dq, 2H), 5.06 (dd, 1H), 5.26 (m, 1H). P(2HB-*co*-3HB-*co*-3HP), δ 1.04 (t, 3H), 1.29 (dd, 3H), 1.93–2.06 (m, 2H), 2.45–2.55 (m, 2H), 2.62–2.68 (m, 2H), 4.32–4.38 (m, 2H), 5.06 (t, 1H), 5.25–5.34 (m, 1H). P(2HB-*co*-3HB-*co*-4H2MB), δ 1.04 (t, 3H), 1.10–1.18 (dd, 3H), 1.28 (d, 3H), 1.91–2.08 (m, 2H), 2.54 (dq, 2H), 4.11 (s, 2H), 5.07 (t, 1H), 5.26 (m, 1H). P(2HB-*co*-3HB-*co*-5HV), δ 1.04 (t, 3H), 1.28 (d, 3H), 1.66 (t, 2H), 1.89–2.08 (m, 2H), 2.22 (t, 2H), 2.54 (dq, 2H), 4.05–4.11 (m, 2H), 5.07 (dd, 1H), 5.26 (m, 1H). P(2HB-*co*-3HB-*co*-6HHx), δ 1.04 (t, 3H), 1.27 (d, 3H), 1.67 (br, 2H), 1.89–2.08 (m, 2H), 2.54 (dq, 2H), 4.03–4.11 (m, 2H), 5.07 (dd, 1H), 5.26 (m, 1H).

The incorporation of LMC units was also verified using GC and GC–MS. Approximately 1 mg polymer dissolved in 500 µL of chloroform was combined with 500 µL of 15:85 (v/v) sulfuric acid/methanol. The mixture was heated at 100 °C for 2 h in a glass tube with a screw cap to convert the polymer into low-molecular-weight derivatives. The reaction product was analyzed, as described previously [62]. The sample concentration was adjusted to approximately 2 mg/mL for GC and diluted to 100 µg/mL for GC–MS.

### Molecular weight measurement of polymers

The average molecular weight and polydispersity of the synthesized polymers were determined by size exclusion chromatography (SEC). Polymers extracted and purified from the *E. coli* cells were dissolved in chloroform to a concentration of 1–5 mg/mL. HPLC system (JASCO, Japan) equipped with two tandem K-806L columns (Shodex, Japan) was used for SEC analysis. The pump flow rate was 0.7 mL/min, the column temperature was 40 °C, and the sample injection volume was 100 μL. The molecular weights of the polymers were calculated from calibration curves prepared using a commercially available polystyrene standard (Shodex).

### Analysis of thermal properties

The glass transition temperature (*T*_g_) and the melting temperature (*T*_m_) of the synthesized polymers were analyzed using DSC. The polymers (5–10 mg) were confined in an aluminum pan using a pressing machine (METTLER TOLEDO™ Crucible Sealing Press). DSC 3 (METTLER TOLEDO) was used as an instrument. To confirm the polymer structure, *T*_m_ was measured after prolonged heating at an isothermal temperature. The measurement was performed under nitrogen atmosphere (flow rate: 100 mL/min) with the following temperature controls: (1) cooling from 30 °C to − 30 °C at 50 °C/min; (2) cooling from − 30 °C to − 50 °C at 20 °C/min; (3) heating from − 50 °C to 210 °C at 20 °C/min; (4) cooling from 210 °C to − 30 °C at 50 °C/min; (5) cooling from − 30 °C to − 50 °C at 20 °C/min; (6) isothermal heating at − 50 °C for 5 min; and (7) heating from − 50 °C to 210 °C at 20 °C/min.

### Solvent fractionation

P(2HB-*co*-3HP) (30 mg) was dissolved in 1 mL of chloroform and incubated at 100 °C for 24 h. The solution was combined with 10 mL of diethylether in small steps, and the mixture was incubated at 4 °C for 3 h. The diethylether-soluble fraction was collected by passing through a PTFE membrane filter, and the polymer on the membrane was collected as the diethylether-insoluble fraction. A blend of P(2HB) and P(3HP) was treated in the same way.

## Supplementary Information

**Additional file 1.** **Fig. S1.** ^1^H-^1^H DOSY-NMR of 4H2MB, 5HV, and 6HHx in D_2_O. **Fig. S2.** GC analysis and Electron Ionization MS spectra of P(3HB-*co*-3HP), P(3HB-*co*-LMC HA)s, and relevant polymers synthesized by PhaC_AR_. **Fig. S3.**^13^C NMR analysis of P(3HB-*co*-LMC HA)s. **Fig. S4A.** ^1^H NMR of P(3HB-*co*- 3HP), P(3HB-*co*-LMC HA)s P(2HB-*co*-3HP), P(2HB-*co*-3HB), P(2HB-*co*-3HB-*co*-3HP), P(2HB-*co*-3HB-*co*-LMC HA)s, and relevant polymers. **Fig. S5.** DSC thermograms of polymers in Table 3.

## Data Availability

All datasets generated and analyzed during this study are included in this published article and its additional files.

## Abbreviations

AlkK: MCL 3HA CoA ligase
CoA: Coenzyme A
DOSY NMR: Diffusion ordered spectroscopy NMR
DSC: Differential scanning calorimetry
GC: Gas chromatography
GL: Glycolate
HA: Hydroxyalkanoate
LA: Lactic acid
LMC: Long-main-chain
MCL: Medium-chain-length
NMR: Nuclear magnetic resonance
PCL: Polycaprolactone
PCT: Propionyl-CoA transferase
PHA: Polyhydroxyalkanoate
PhaC: PHA synthase
PLA: Polylactic acid
PTFE: Polytetrafluoroethylene
ROP: Ring-opening polymerization
SCL: Short-chain-length
SEC: Size exclusion chromatography
*T*_g_: Glass transition temperature
*T*_m_: Melting temperature
2HB: 2-Hydroxybutyrate
3HB: 3-Hydroxybutyrate
3HHx: 3-Hydroxyhexanoate
3HP: 3-Hydroxypropionate
4HB: 4-Hydroxybutyrate
4H2MB: 4-Hydroxy-2-methylbutyrate
5HV: 5-Hydroxyvalerate
6HHx: 6-Hydroxyhexanoate

## References

[1] Nakayama A, Yamano N, Kawasaki N (2019). Biodegradation in seawater of aliphatic polyesters. Polym Degrad Stab.

[2] Suzuki M, Tachibana Y, Kasuya K (2021). Biodegradability of poly(3-hydroxyalkanoate) and poly(ε-caprolactone) via biological carbon cycles in marine environments. Polym J.

[3] Narancic T, Verstichel S, Reddy Chaganti S, Morales-Gamez L, Kenny ST, De Wilde B, Babu Padamati R, O'Connor KE (2018). Biodegradable plastic blends create new possibilities for end-of-life management of plastics but they are not a panacea for plastic pollution. Environ Sci Technol.

[4] Folino A, Karageorgiou A, Calabro PS, Komilis D (2020). Biodegradation of wasted bioplastics in natural and industrial environments: A review. Sustainability.

[5] Lofgren A, Albertsson AC, Dubois P, Jerome R (1995). Recent advances in ring-opening polymerization of lactones and related-compounds. J Macromol Sci Rev Macromol Chem Phys.

[6] Kobayashi S (2009). Recent developments in lipase-catalyzed synthesis of polyesters. Macromol Rapid Commun.

[7] Albertsson AC, Srivastava RK (2008). Recent developments in enzyme-catalyzed ring-opening polymerization. Adv Drug Del Rev.

[8] Rehm BHA (2003). Polyester synthases: natural catalysts for plastics. Biochem J.

[9] Vigneswari S, Noor MSM, Amelia TSM, Balakrishnan K, Adnan A, Bhubalan K, Amirul AA, Ramakrishna S (2021). Recent advances in the biosynthesis of polyhydroxyalkanoates from lignocellulosic feedstocks. Life (Basel)..

[10] Marciniak P, Mozejko-Ciesielska J (2021). What is new in the field of industrial wastes conversion into polyhydroxyalkanoates by bacteria?. Polymers (Basel).

[11] Sudesh K, Abe H, Doi Y (2000). Synthesis, structure and properties of polyhydroxyalkanoates: biological polyesters. Prog Polym Sci.

[12] Arikawa H, Sato S, Fujiki T, Matsumoto K (2016). A study on the relation between poly(3-hydroxybutyrate) depolymerases or oligomer hydrolases and molecular weight of polyhydroxyalkanoates accumulating in *Cupriavidus necator* H16. J Biotechnol.

[13] Egusa EA, Edwards DJ, Thao ML, Kirk LL, Hanne LF (2018). Isolation and characterization of bacteria that produce polyhydroxybutyrate depolymerases. J Microbiol Biol Educ.

[14] Budurova D, Ublekov F, Penchev H (2021). The use of formic acid as a common solvent for electrospinning of hybrid PHB/Soy protein fibers. Mater Lett.

[15] Rofeal M, Abd El-Malek F, Qi XH (2021). In vitro assessment of green polyhydroxybutyrate/chitosan blend loaded with kaempferol nanocrystals as a potential dressing for infected wounds. Nanotechnology.

[16] Danko M, Mosnackova K, Vykydalova A, Kleinova A, Puskarova A, Pangallo D, Bujdos M, Mosnacek J (2021). Properties and degradation performances of biodegradable poly(lactic acid)/poly(3-hydroxybutyrate) blends and keratin composites. Polymers.

[17] Orita I, Unno G, Kato R, Fukui T (2022). Biosynthesis of polyhydroxyalkanoate terpolymer from methanol via the reverse β-oxidation pathway in the presence of lanthanide. Microorganisms.

[18] Sato S, Maruyama H, Fujiki T, Matsumoto K (2015). Regulation of 3-hydroxyhexanoate composition in PHBH synthesized by recombinant *Cupriavidus necator* H16 from plant oil by using butyrate as a *co*-substrate. J Biosci Bioeng.

[19] Doi Y, Kitamura S, Abe H (1995). Microbial synthesis and characterization of poly(3-hydroxybutyrate-*co*-3-hydroxyhexanoate). Macromolecules.

[20] Matsusaki H, Abe H, Doi Y (2000). Biosynthesis and properties of poly(3-hydroxybutyrate-*co*-3-hydroxyalkanoates) by recombinant strains of *Pseudomonas* sp. 61–3. Biomacromol.

[21] Liu CH, Chen HY, Chen YL, Sheu DS (2021). The polyhydroxyalkanoate (PHA) synthase 1 of Pseudomonas sp H9 synthesized a 3-hydroxybutyrate-dominant hybrid of short- and medium-chain-length PHA. Enzyme Microb Technol.

[22] Nomura CT, Taguchi S (2007). PHA synthase engineering toward superbiocatalysts for custom-made biopolymers. Appl Microbiol Biotechnol.

[23] Taguchi S, Yamada M, Matsumoto K, Tajima K, Satoh Y, Munekata M, Ohno K, Kohda K, Shimamura T, Kambe H, Obata S (2008). A microbial factory for lactate-based polyesters using a lactate-polymerizing enzyme. Proc Natl Acad Sci U S A.

[24] Chek MF, Hiroe A, Hakoshima T, Sudesh K, Taguchi S (2019). PHA synthase (PhaC): interpreting the functions of bioplastic-producing enzyme from a structural perspective. Appl Microbiol Biotechnol.

[25] McCool GJ, Cannon MC (2001). PhaC and PhaR are required for polyhydroxyalkanoic acid synthase activity in *Bacillus megaterium*. J Bacteriol.

[26] Matsumoto K, Ishiyama A, Sakai K, Shiba T, Taguchi S (2011). Biosynthesis of glycolate-based polyesters containing medium-chain-length 3-hydroxyalkanoates in recombinant *Escherichia coli* expressing engineered polyhydroxyalkanoate synthase. J Biotechnol.

[27] Matsumoto K, Terai S, Ishiyama A, Sun J, Kabe T, Song Y, Nduko JM, Iwata T, Taguchi S (2013). One-pot microbial production, mechanical properties and enzymatic degradation of isotactic P[(*R*)-2-hydroxybutyrate] and its copolymer with (*R*)-lactate. Biomacromol.

[28] Park SJ, Lee TW, Lim SC, Kim TW, Lee H, Kim MK, Lee SH, Song BK, Lee SY (2012). Biosynthesis of polyhydroxyalkanoates containing 2-hydroxybutyrate from unrelated carbon source by metabolically engineered *Escherichia coli*. Appl Microbiol Biotechnol.

[29] Mizuno S, Enda Y, Saika A, Hiroe A, Tsuge T (2018). Biosynthesis of polyhydroxyalkanoates containing 2-hydroxy-4-methylvalerate and 2-hydroxy-3-phenylpropionate units from a related or unrelated carbon source. J Biosci Bioeng.

[30] Goto S, Hokamura A, Shiratsuchi H, Taguchi S, Matsumoto K, Abe H, Tanaka K, Matsusaki H (2019). Biosynthesis of novel lactate-based polymers containing medium-chain-length 3-hydroxyalkanoates by recombinant *Escherichia coli* strains from glucose. J Biosci Bioeng.

[31] Feng HB, Lu XY, Wang WY, Kang NG, Mays JW (2017). Block copolymers: Synthesis, self-assembly, and applications. Polymers.

[32] Tripathi L, Wu LP, Meng D, Chen J, Chen GQ (2013). Biosynthesis and characterization of diblock copolymer of P(3-hydroxypropionate)-*block*-P(4-hydroxybutyrate) from recombinant *Escherichia coli*. Biomacromol.

[33] Matsumoto K, Hori C, Fujii R, Takaya M, Ooba T, Ooi T, Isono T, Satoh T, Taguchi S (2018). Dynamic changes of intracellular monomer levels regulate block sequence of polyhydroxyalkanoates in engineered *Escherichia coli*. Biomacromol.

[34] Matsumoto K, Takase K, Yamamoto Y, Doi Y, Taguchi S (2009). Chimeric enzyme composed of polyhydroxyalkanoate (PHA) synthases from *Ralstonia eutropha* and *Aeromonas caviae* enhances production of PHAs in recombinant *Escherichia coli*. Biomacromol.

[35] Arai S, Sakakibara S, Mareschal R, Ooi T, Zinn M, Matsumoto K (2020). Biosynthesis of random-homo block copolymer poly[glycolate-*ran*-3-hydroxybutyrate (3HB)]-*b*-poly(3HB) using sequence-regulating chimeric polyhydroxyalkanoate synthase in *Escherichia coli*. Front Bioeng Biotechnol.

[36] Tomita H, Satoh K, Nomura CT, Matsumoto K (2022). Biosynthesis of poly(glycolate-*co*-3-hydroxybutyrate-*co*-3-hydroxyhexanoate) in *Escherichia coli* expressing sequence-regulating polyhydroxyalkanoate synthase and medium-chain-length 3-hydroxyalkanoic acid coenzyme A ligase. Biosci Biotechnol Biochem.

[37] Kageyama Y, Tomita H, Isono T, Satoh T, Matsumoto K (2021). Artificial polyhydroxyalkanoate poly[2-hydroxybutyrate-*block*-3-hydroxybutyrate] elastomer-like material. Sci Rep.

[38] Utsunomia C, Ren Q, Zinn M (2020). Poly(4-hydroxybutyrate): Current state and perspectives. Front Bioeng Biotechnol.

[39] Houk KN, Jabbari A, Hall HK, Aleman C (2008). Why δ-valerolactone polymerizes and γ-butyrolactone does not. J Org Chem.

[40] Chuah JA, Yamada M, Taguchi S, Sudesh K, Doi Y, Numata K (2013). Biosynthesis and characterization of polyhydroxyalkanoate containing 5-hydroxyvalerate units: Effects of 5HV units on biodegradability, cytotoxicity, mechanical and thermal properties. Polym Degrad Stab.

[41] Myung J, Flanagan JCA, Waymouth RM, Criddle CS (2017). Expanding the range of polyhydroxyalkanoates synthesized by methanotrophic bacteria through the utilization of ω-hydroxyalkanoate co-substrates. AMB Express.

[42] Abe H, Doi Y, Aoki H, Akehata T, Hori Y, Yamaguchi A (1995). Physical-properties and enzymatic degradability of copolymers of (*R*)-3-hydroxybutyric and 6-hydroxyhexanoic acids. Macromolecules.

[43] Hori Y, Takahashi Y, Yamaguchi A, Hagiwara T (1995). Chemical synthesis of novel biodegradable polyesters. Can J Microbiol.

[44] Andreessen B, Taylor N, Steinbuchel A (2014). Poly(3-hydroxypropionate): a promising alternative to fossil fuel-based materials. Appl Environ Microbiol.

[45] Sato S, Andreessen B, Steinbuchel A (2015). Strain and process development for poly(3HB-*co*-3HP) fermentation by engineered *Shimwellia blattae* from glycerol. AMB Express.

[46] McGregor C, Minton NP, Kovacs K (2021). Biosynthesis of poly(3HB-*co*-3HP) with variable monomer composition in recombinant *Cupriavidus necator* H16. ACS Synth Biol.

[47] Dunn EW, Lamb JR, LaPointe AM, Coates GW (2016). Carbonylation of ethylene oxide to β-propiolactone: A facile route to poly(3-hydroxypropionate) and acrylic acid. ACS Catal.

[48] Spaninger E, Bren U (2020). Carcinogenesis of β-propiolactone: A computational study. Chem Res Toxicol.

[49] Andreessen B, Steinbuchel A (2010). Biosynthesis and biodegradation of 3-hydroxypropionate-containing polyesters. Appl Environ Microbiol.

[50] Rehm BHA, Krüger N, Steinbüchel A (1998). A new metabolic link between fatty acid de novo synthesis and polyhydroxyalkanoic acid synthesis. The *phaG* gene from *Pseudomonas putida* KT2440 encodes a 3-hydroxyacyl-acyl carrier protein-coenzyme a transferase. J Biol Chem.

[51] Wang Q, Tappel RC, Zhu C, Nomura CT (2012). Development of a new strategy for production of medium-chain-length polyhydroxyalkanoates by recombinant *Escherichia coli* via inexpensive non-fatty acid feedstocks. Appl Environ Microbiol.

[52] Han X, Satoh Y, Satoh T, Matsumoto K, Kakuchi T, Taguchi S, Dairi T, Munekata M, Tajima K (2011). Chemo-enzymatic synthesis of polyhydroxyalkanoate (PHA) incorporating 2-hydroxybutyrate by wild-type class I PHA synthase from *Ralstonia eutropha*. Appl Microbiol Biotechnol.

[53] Wong YM, Brigham CJ, Rha C, Sinskey AJ, Sudesh K (2012). Biosynthesis and characterization of polyhydroxyalkanoate containing high 3-hydroxyhexanoate monomer fraction from crude palm kernel oil by recombinant *Cupriavidus necator*. Bioresour Technol.

[54] Volova TG, Syrvacheva DA, Zhila NO, Sukovatiy AG (2016). Synthesis of P(3HB-*co*-3HHx) copolymers containing high molar fraction of 3-hydroxyhexanoate monomer by *Cupriavidus eutrophus* B10646. J Chem Technol Biotechnol.

[55] Mukai K, Doi Y, Sema Y, Tomita K (1993). Substrate specificities in hydrolysis of polyhydroxyalkanoates by microbial esterases. Biotechnol Lett.

[56] Saito Y, Doi Y (1994). Microbial synthesis and properties of poly(3-hydroxybutyrate-*co*-4-hydroxybutyrate) in *Comamonas acidovorans*. Int J Biol Macromol.

[57] Mandic M, Spasic J, Ponjavic M, Nikolic MS, Cosovic VR, O'Connor KE, Nikodinovic-Runic J, Djokic L, Jeremic S (2019). Biodegradation of poly(ε-caprolactone) (PCL) and medium chain length polyhydroxyalkanoate (mcl-PHA) using whole cells and cell free protein preparations of *Pseudomonas* and *Streptomyces* strains grown on waste cooking oil. Polym Degrad Stab.

[58] Sekiguchi T, Saika A, Nomura K, Watanabe T, Watanabe T, Fujimoto Y, Enoki M, Sato T, Kato C, Kanehiro H (2011). Biodegradation of aliphatic polyesters soaked in deep seawaters and isolation of poly(ε-caprolactone)-degrading bacteria. Polym Degrad Stab.

[59] Buckley RM, Stubbe J (2015). Chemistry with an artificial primer of polyhydroxybutyrate synthase suggests a mechanism for chain termination. Biochemistry.

[60] Takase K, Taguchi S, Doi Y (2003). Enhanced synthesis of poly(3-hydroxybutyrate) in recombinant *Escherichia coli* by means of error-prone PCR mutagenesis, saturation mutagenesis, and *in vitro* recombination of the type II polyhydroxyalkanoate synthase gene. J Biochem.

[61] Phan HT, Hosoe Y, Guex M, Tomoi M, Tomita H, Zinn M, Matsumoto K (2022). Directed evolution of sequence-regulating polyhydroxyalkanoate synthase to synthesize a medium-chain-length-short-chain-length (MCL-SCL) block copolymer. Biomacromol.

[62] Kato M, Bao HJ, Kang CK, Fukui T, Doi Y (1996). Production of a novel copolyester of 3-hydroxybutyric acid and medium chain length 3-hydroxyalkanaic acids by *Pseudomonas* sp. 61–3 from sugars. Appl Microbiol Biotechnol.

