# Supplementary material for: Versatile aliphatic polyester biosynthesis system for producing random and block copolymers composed of 2-, 3-, 4-, 5-, and 6-hydroxyalkanoates using the sequence-regulating polyhydroxyalkanoate synthase PhaCAR
Source: Microb Cell Fact. 2022 May 14;21:84. doi: 10.1186/s12934-022-01811-7 (PMC9107728; doi:10.1186/s12934-022-01811-7)
Supplement: Supplementary file 1 — Additional file 1. Fig. S1. 1H-1H DOSY-NMR of 4H2MB, 5HV, and 6HHx in D2O. Fig. S2. GC analysis and Electron Ionization MS spectra of P(3HB-co-3HP), P(3HB-co-LMC HA)s, and relevant polymers synthesized by PhaCAR. Fig. S3.13C NMR analysis of P(3HB-co-LMC HA)s. Fig. S4A. 1H NMR of P(3HB-co- 3HP), P(3HB-co-LMC HA)s P(2HB-co-3HP), P(2HB-co-3HB), P(2HB-co-3HB-co-3HP), P(2HB-co-3HB-co-LMC HA)s, and relevant polymers. Fig. S5. DSC thermograms of polymers in Table 3. [file 12934_2022_1811_MOESM1_ESM.docx]

*Supporting information*

*to*

**Versatile aliphatic polyester biosynthesis system for producing random and block copolymers composed of 2-, 3-, 4-, 5-, and 6-hydroxyalkanoates using the sequence-regulating polyhydroxyalkanoate synthase PhaC_AR_**

Keigo Satoh, Tomoya Kawakami, Nagi Isobe, Loïc Pasquier,

Hiroya Tomita, Manfred Zinn, and Ken’ichiro Matsumoto


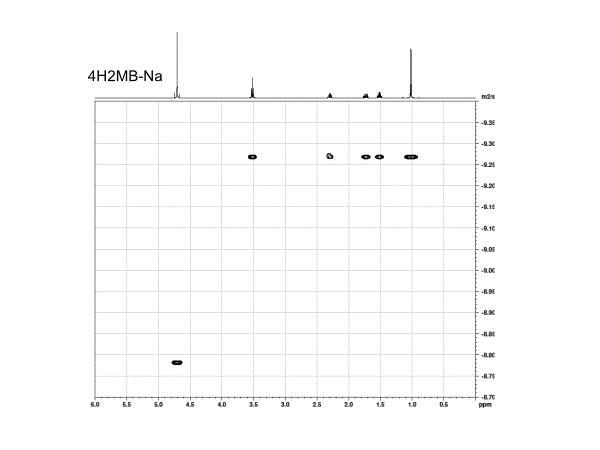


^1^H [log(m^2^/s)]

**Fig. S1A** ^1^H-^1^H DOSY-NMR of 4H2MB-Na in D_2_O


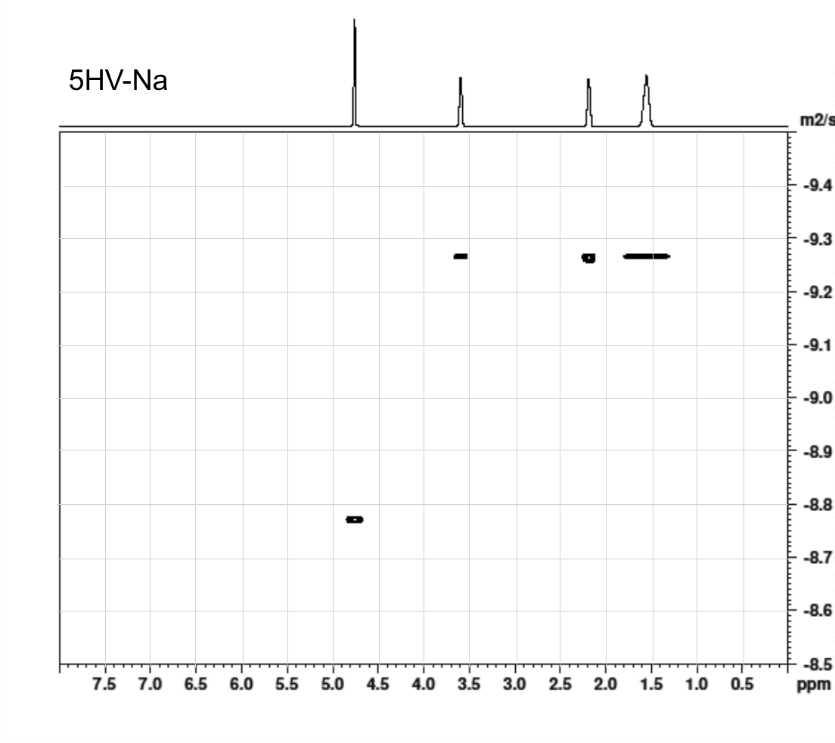


^1^H [log(m^2^/s)]

**Fig. S1B** ^1^H-^1^H DOSY-NMR of 5HV-Na in D_2_O


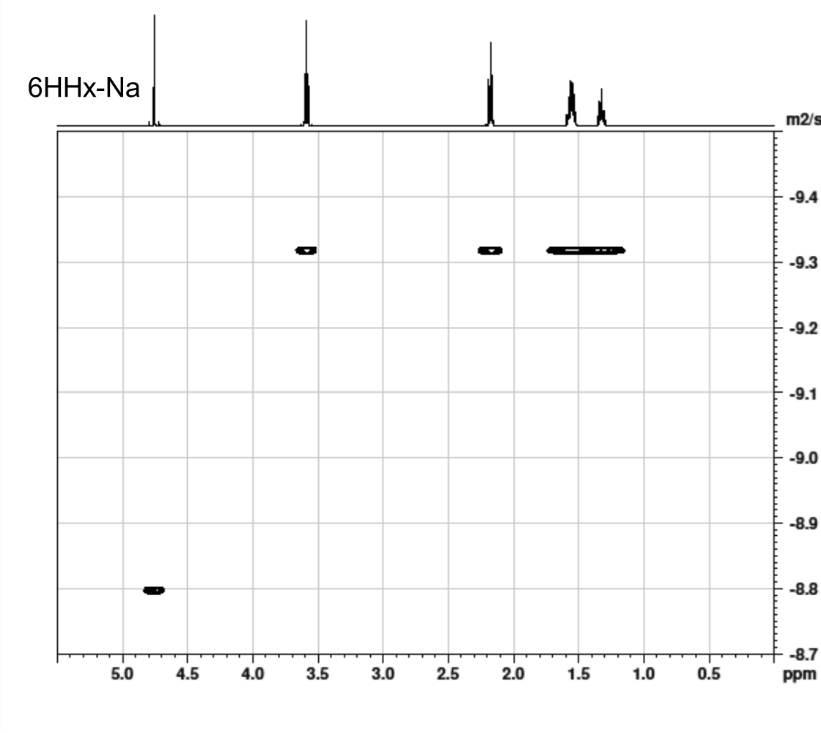


^1^H [log(m^2^/s)]

**Fig. S1C** ^1^H-^1^H DOSY-NMR of 6HHx-Na in D_2_O


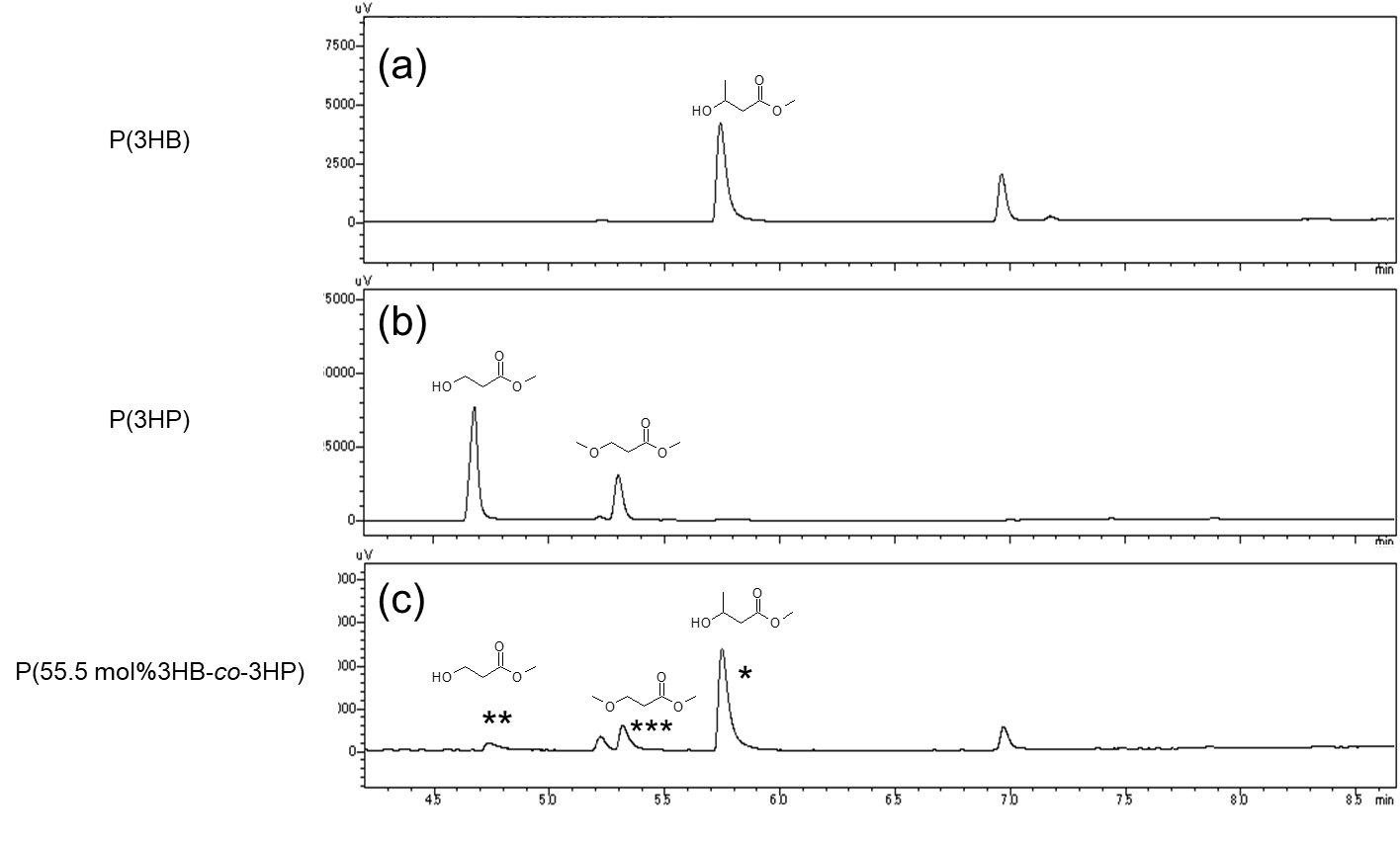


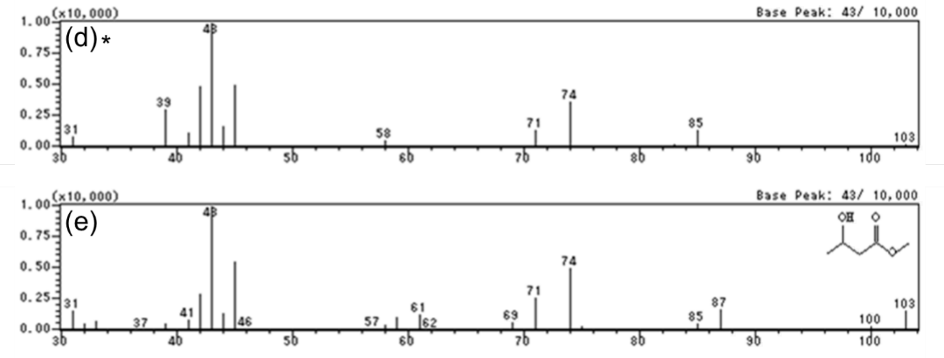


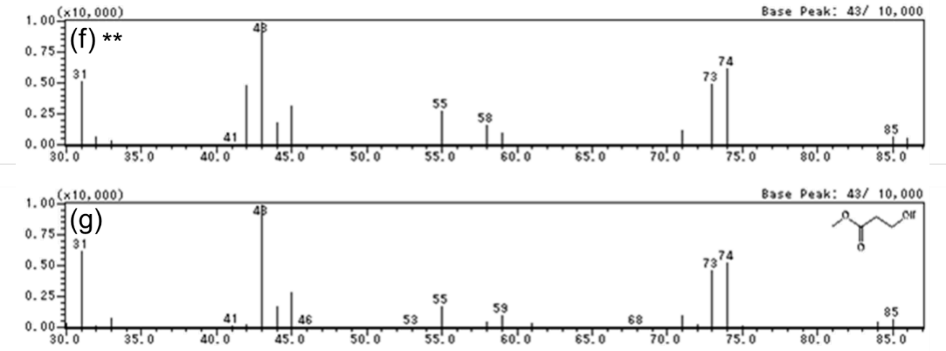


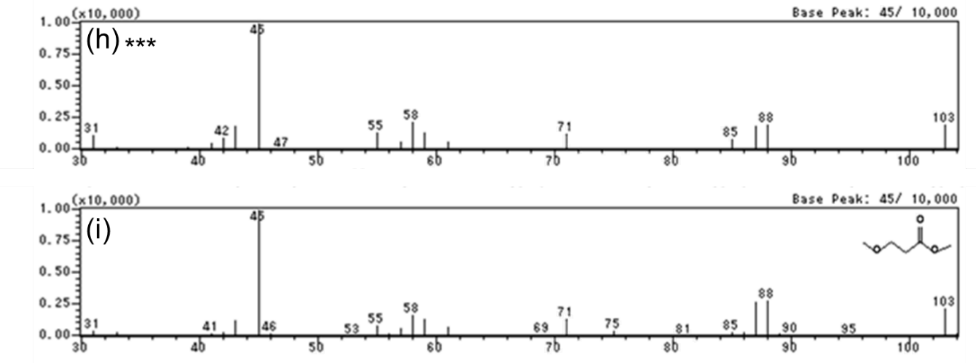


**Fig. S2A** GC analysis and Electron Ionization (EI) MS spectra of P(3HB), P(3HP) and P(3HB-*co*­-3HP) synthesized by PhaC_AR_**.** GC charts of P(3HB) (a), P(3HP) (b) and P(3HB-*co*­-3HP) (c) methanolysis derivatives. MS spectra corresponding to peak * (d), methyl 3-hydroxybutyrate standard in NIST library (e), peak ** (f), methyl 3-hydroxypropionate standard (g), peak *** (h) and methyl 3-methoxypropionate standard (i).

_
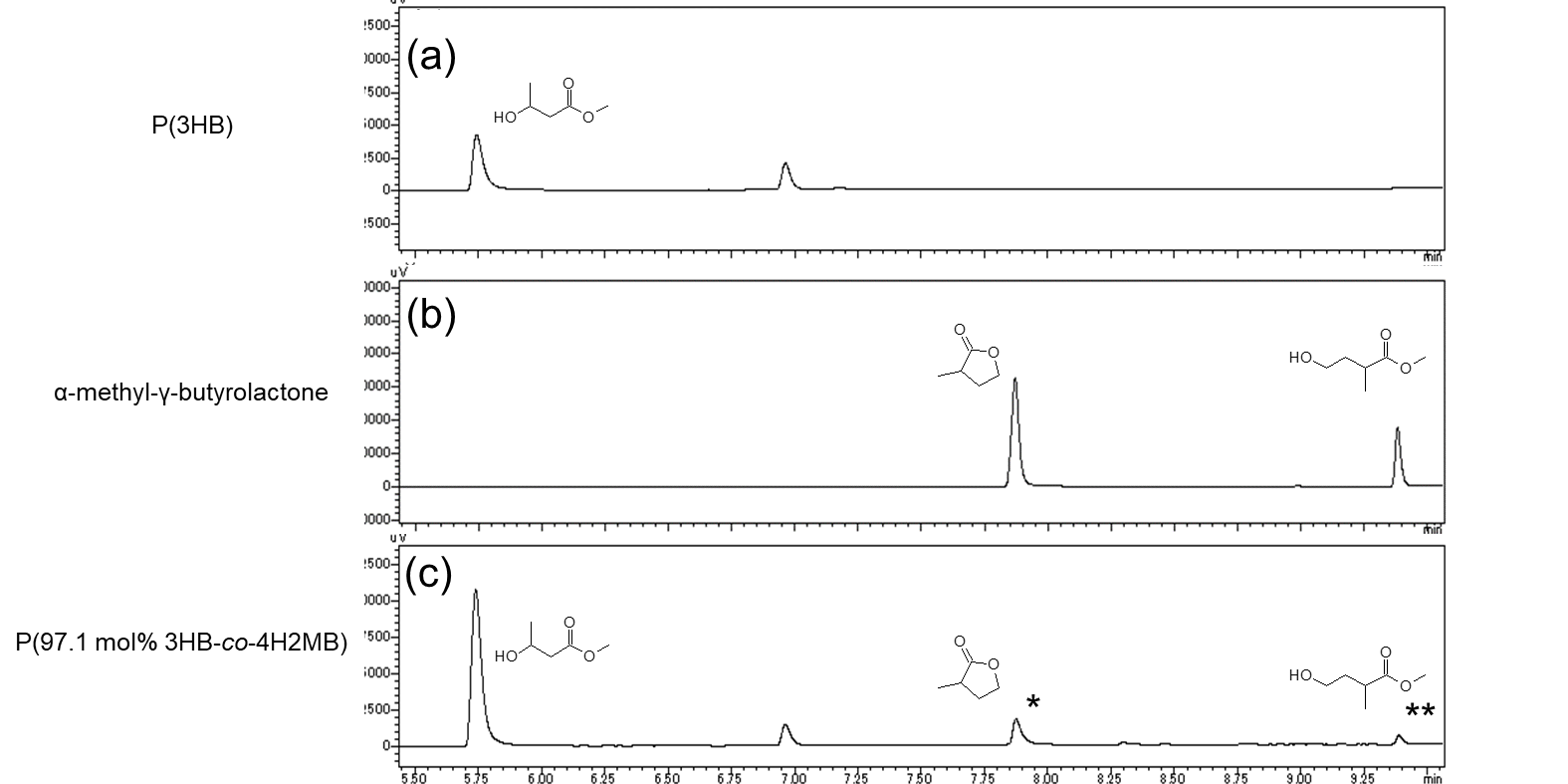
_


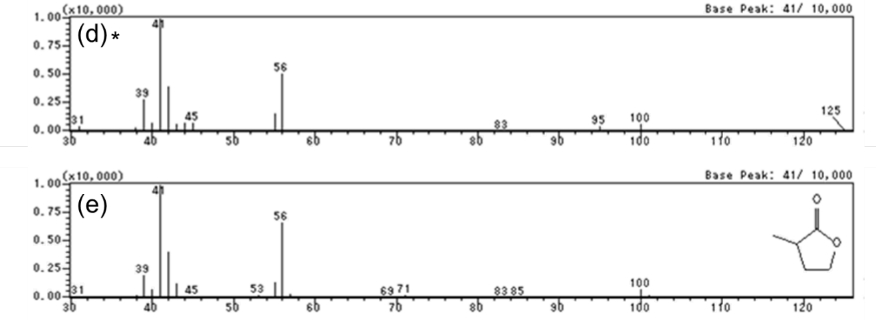


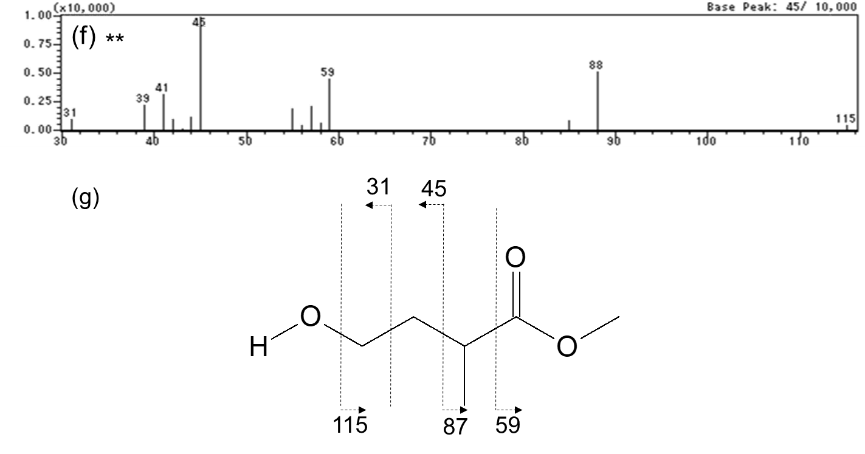


**Fig. S2B** GC analysis and MS spectra of P(3HB-*co*­-4H2MB) synthesized by PhaC_AR_. GC charts of P(3HB) (a), α-methyl-γ-butyrolactone (b) and P(3HB-*co*­-4H2MB) (c). MS spectra of peak * (d), α-methyl-γ-butyrolactone standard (e) and peak **(f). The fragment m/z pattern generated from methyl 2-methyl-4-hydroxybutyrate (g).

**
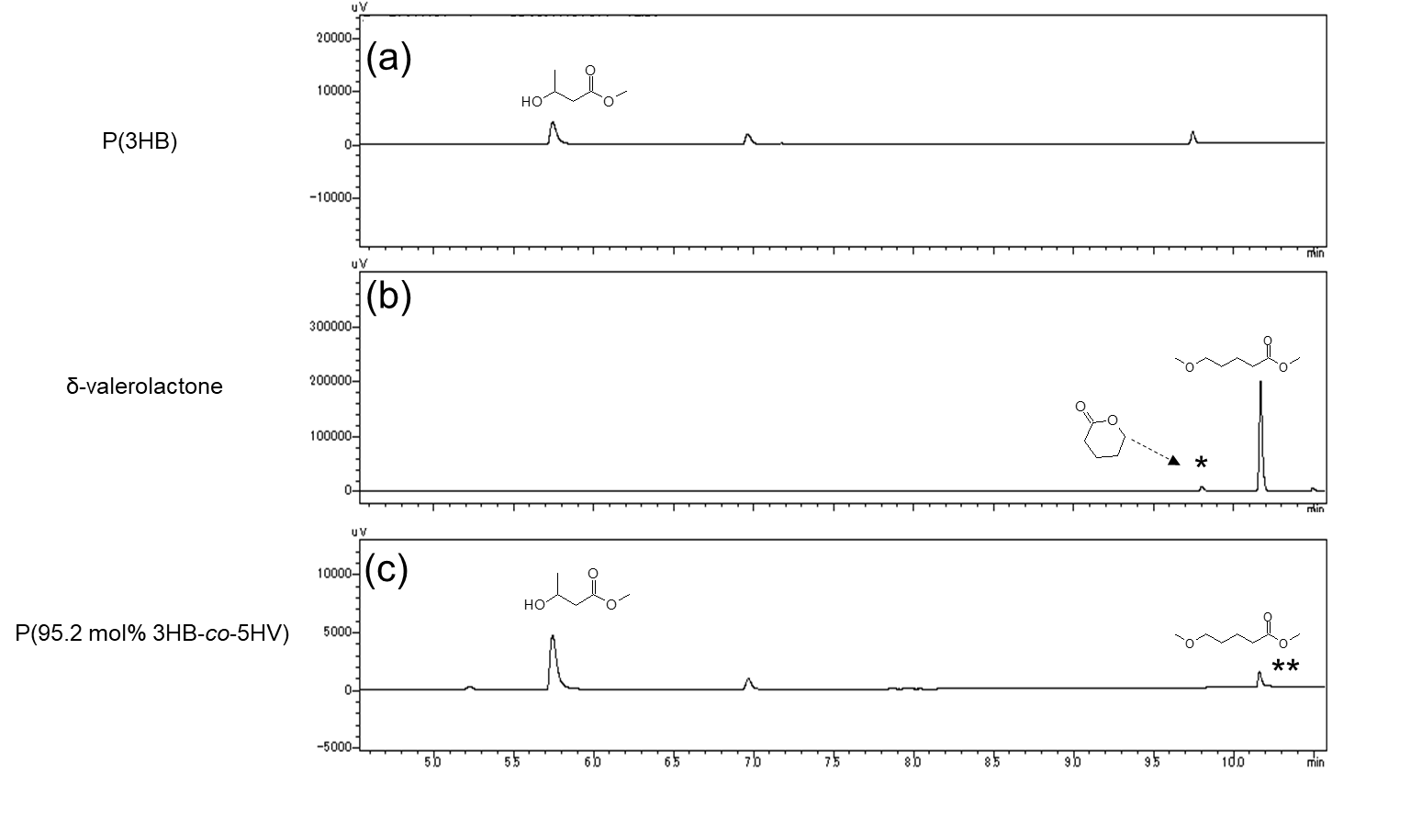
**

**
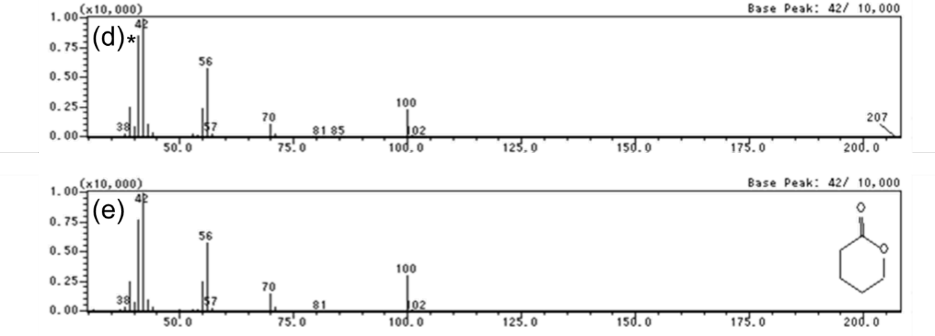
**

**
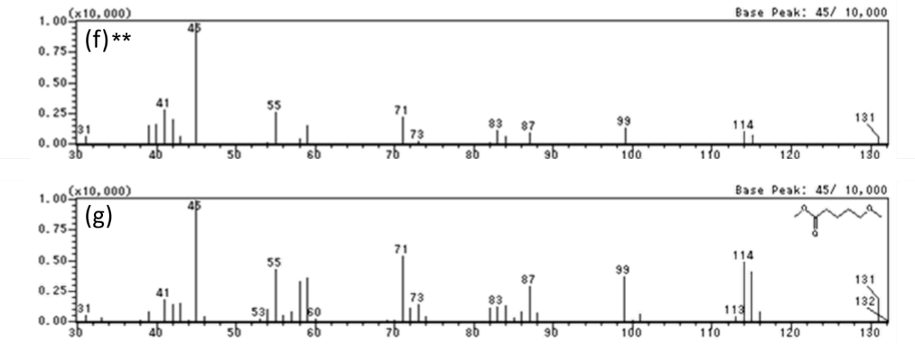
**

**Fig. S2C** GC analysis and MS spectra of P(3HB-*co*­-5HV) synthesized by PhaC_AR_. GC charts of P(3HB) (a), δ-valerolactone (b) and P(3HB-*co*-5HV) (c). MS spectra of peak * (d), δ-valerolactone standard (e), peak ** (f) and methyl 5-methoxypentanoate (g).


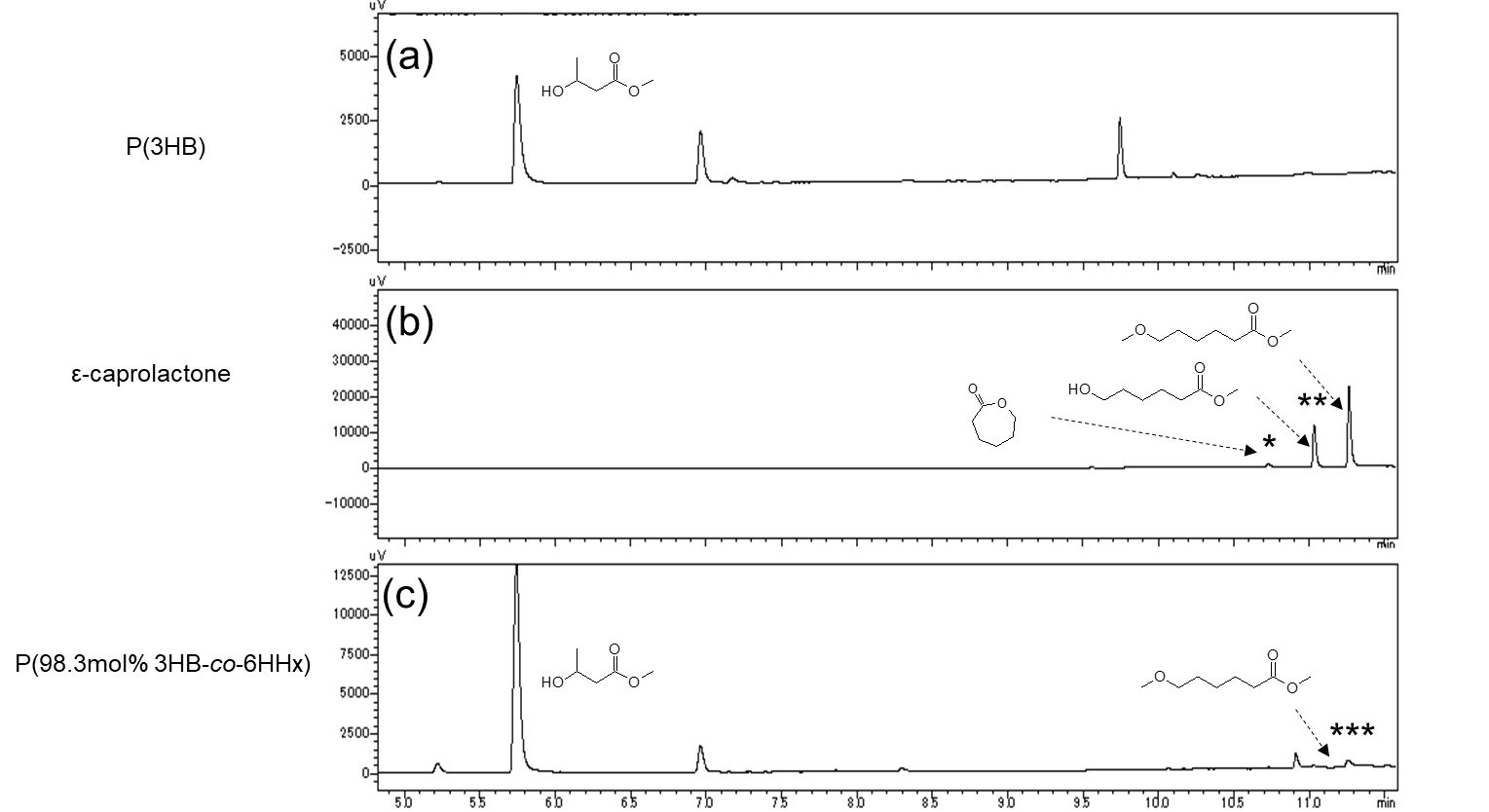


**
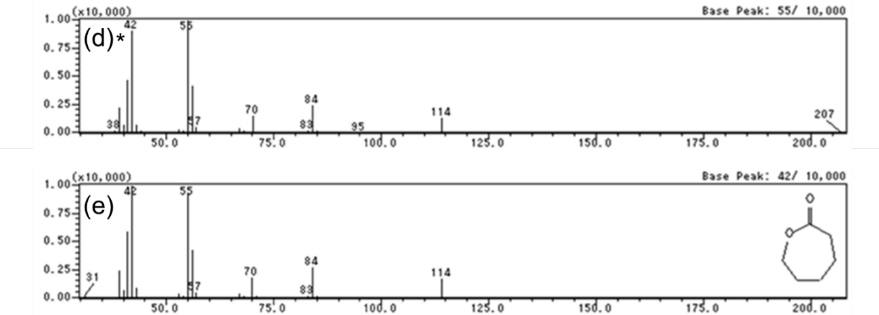
**


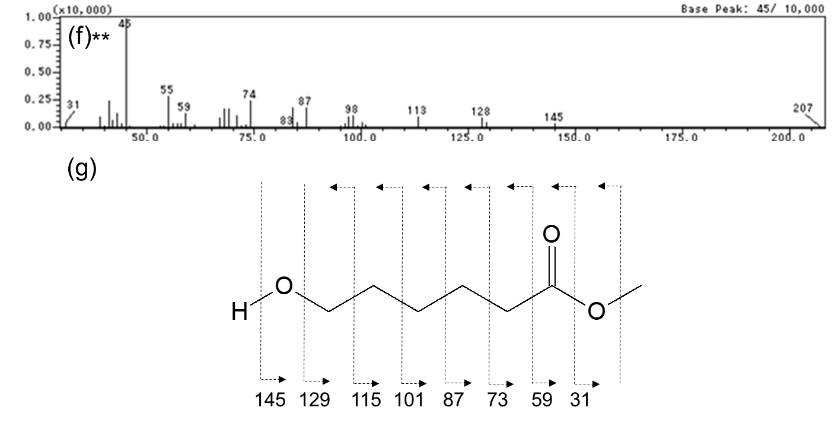


**
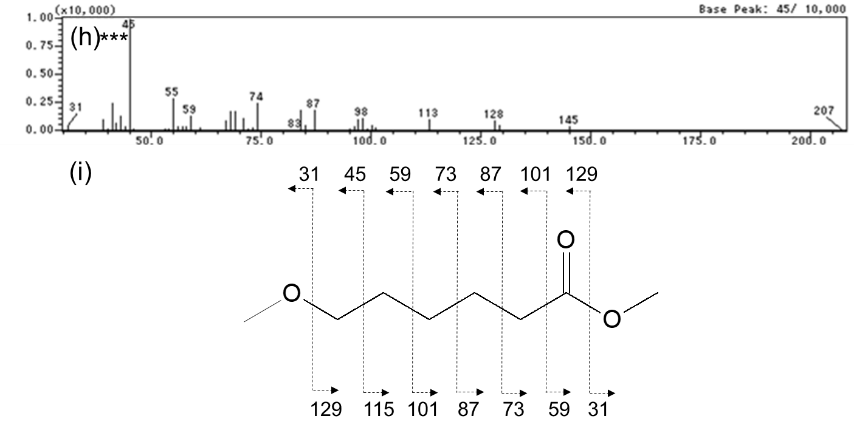
**

**Fig. S2D** GC analysis and MS spectra of P(3HB-*co*­-6HHx) synthesized by PhaC_AR_

GC charts of P(3HB) (a), ε-caprolactone (b) and P(3HB-*co*-6HHx) (c). MS spectra of peak * (d), methyl 6-methoxyhexanoate standard (e) and peak ** (f). The fragment m/z pattern generated from methyl 6-hydroxyhexanoate (g). MS spectrum of peak *** (h). The fragment m/z pattern generated from methyl 6-methoxyhexanoate (i).


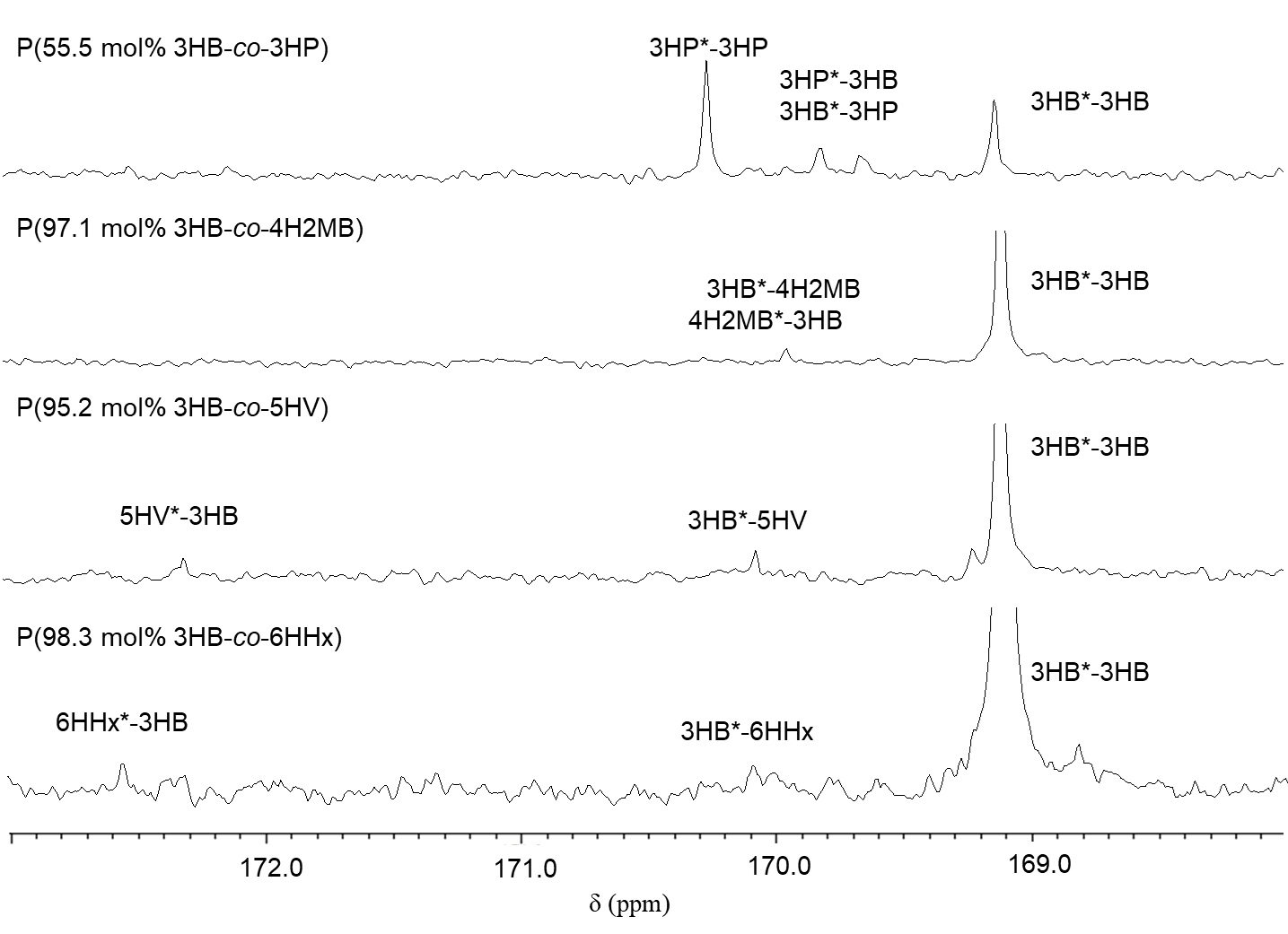


**Fig. S3** ^13^C NMR analysis of P(3HB-*co*-LMC HA)s. Resonances of carbons of carbonyl group are magnified. The dyad sequences of P(3HB-*co*-6HHx) are ascribed based on the reference No. 42. The resonances of 4H2MB and 5HV containing copolymers are ascribed based on the analogy to P(3HB-*co*-6HHx).


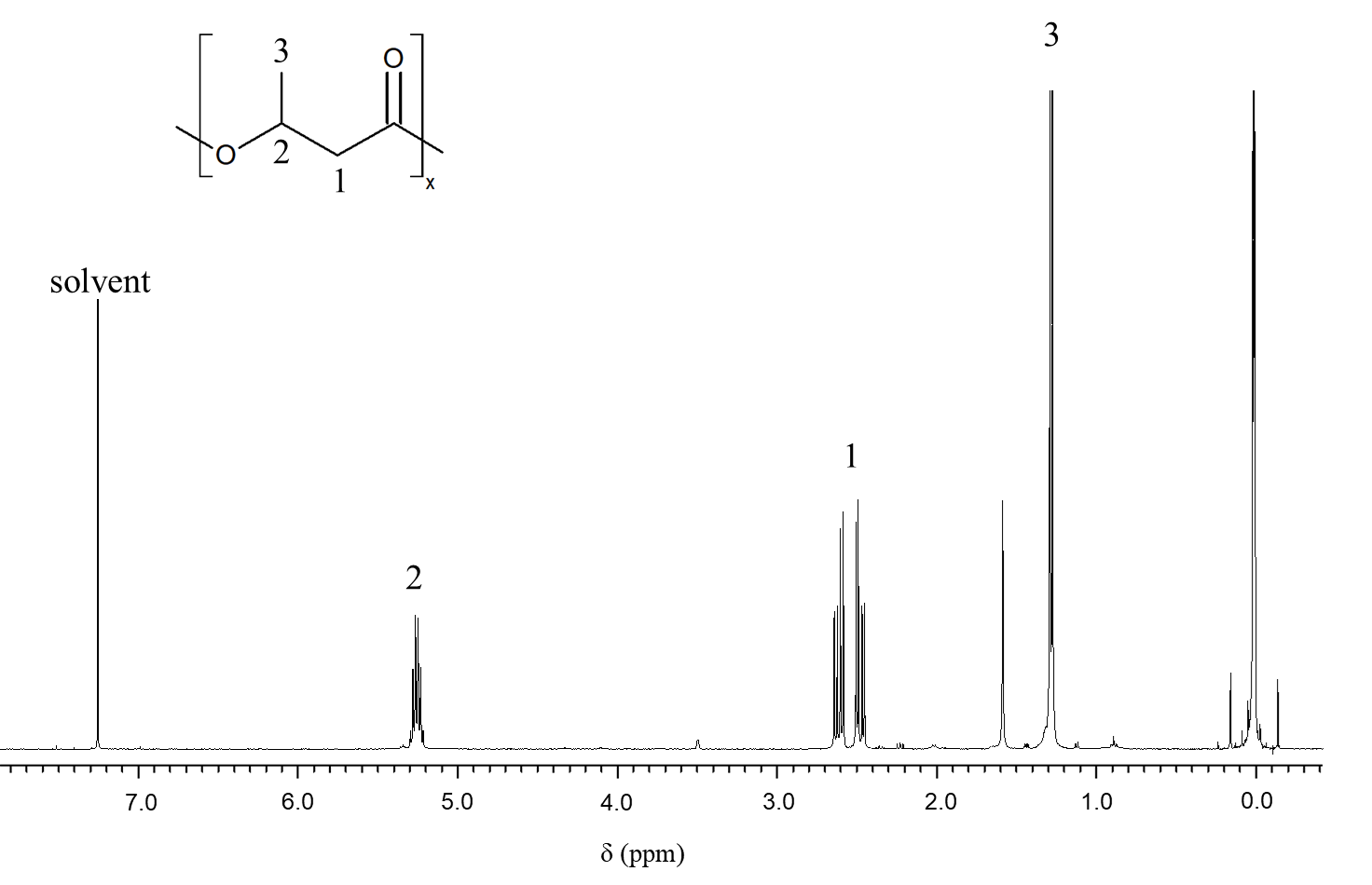


**Fig. S4A** ^１^H NMR of P(3HB)


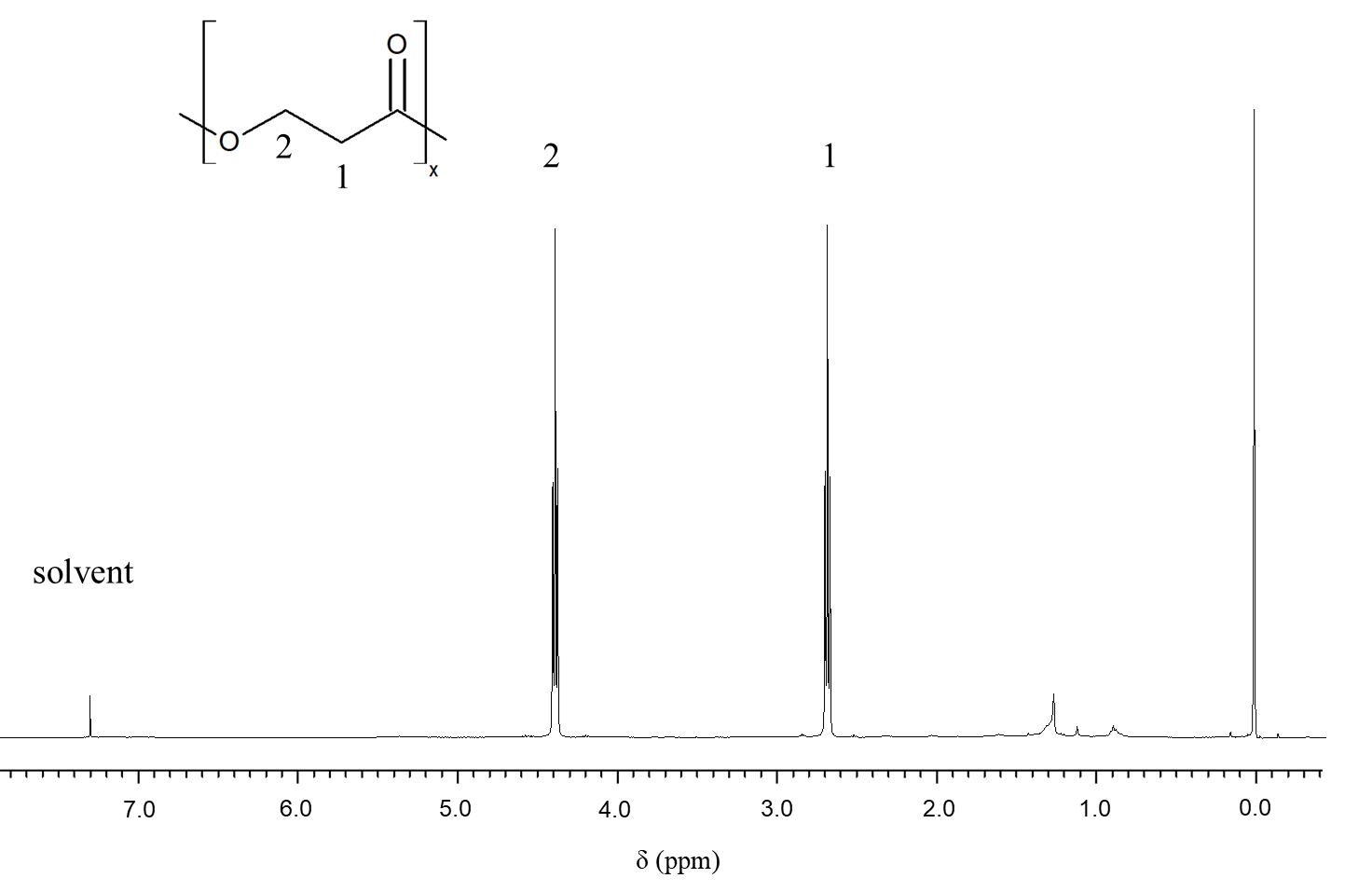


**Fig. S4B** ^１^H NMR of P(3HP)


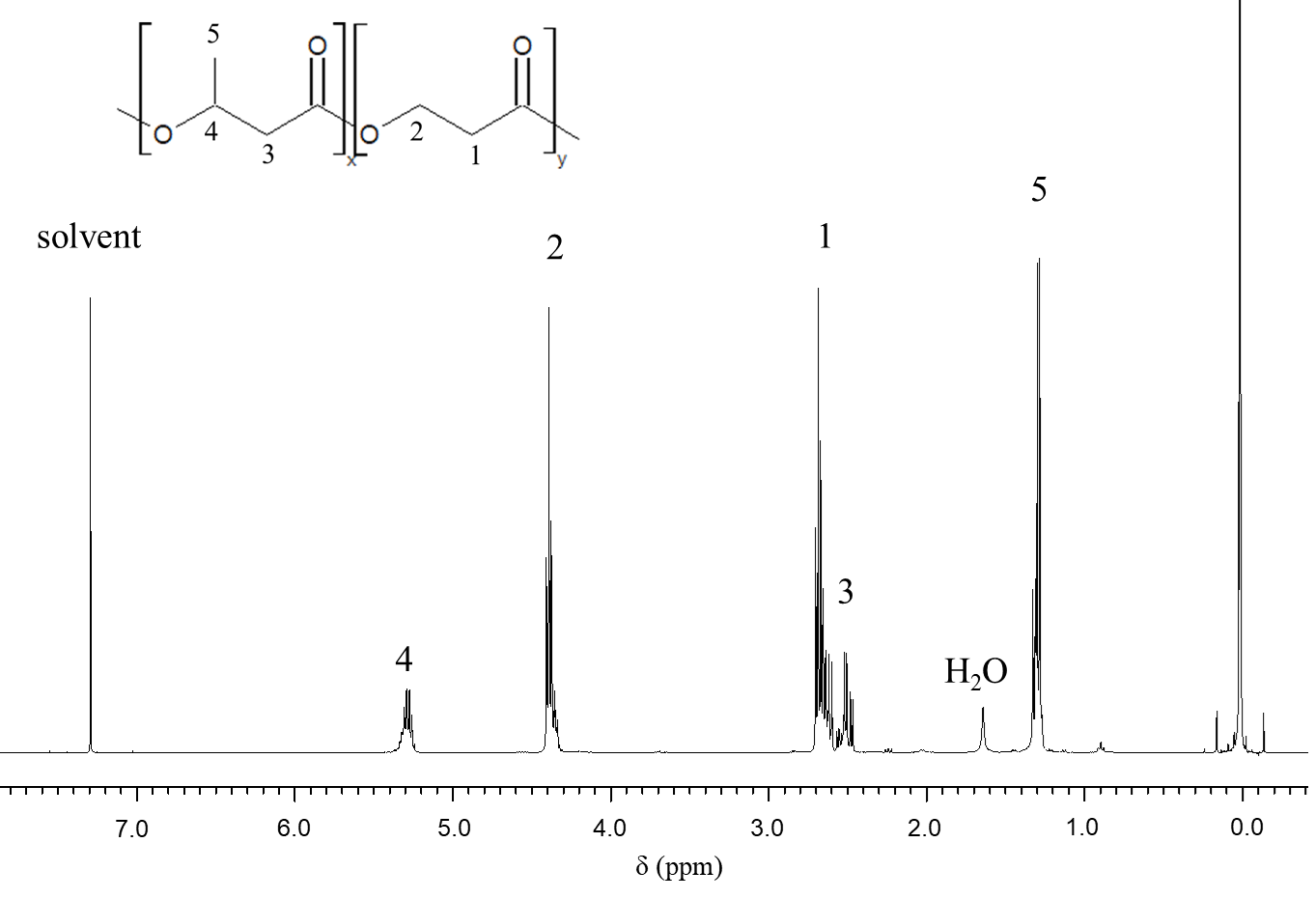


**Fig. S4C** ^１^H NMR of P(3HB-*co*-3HP)


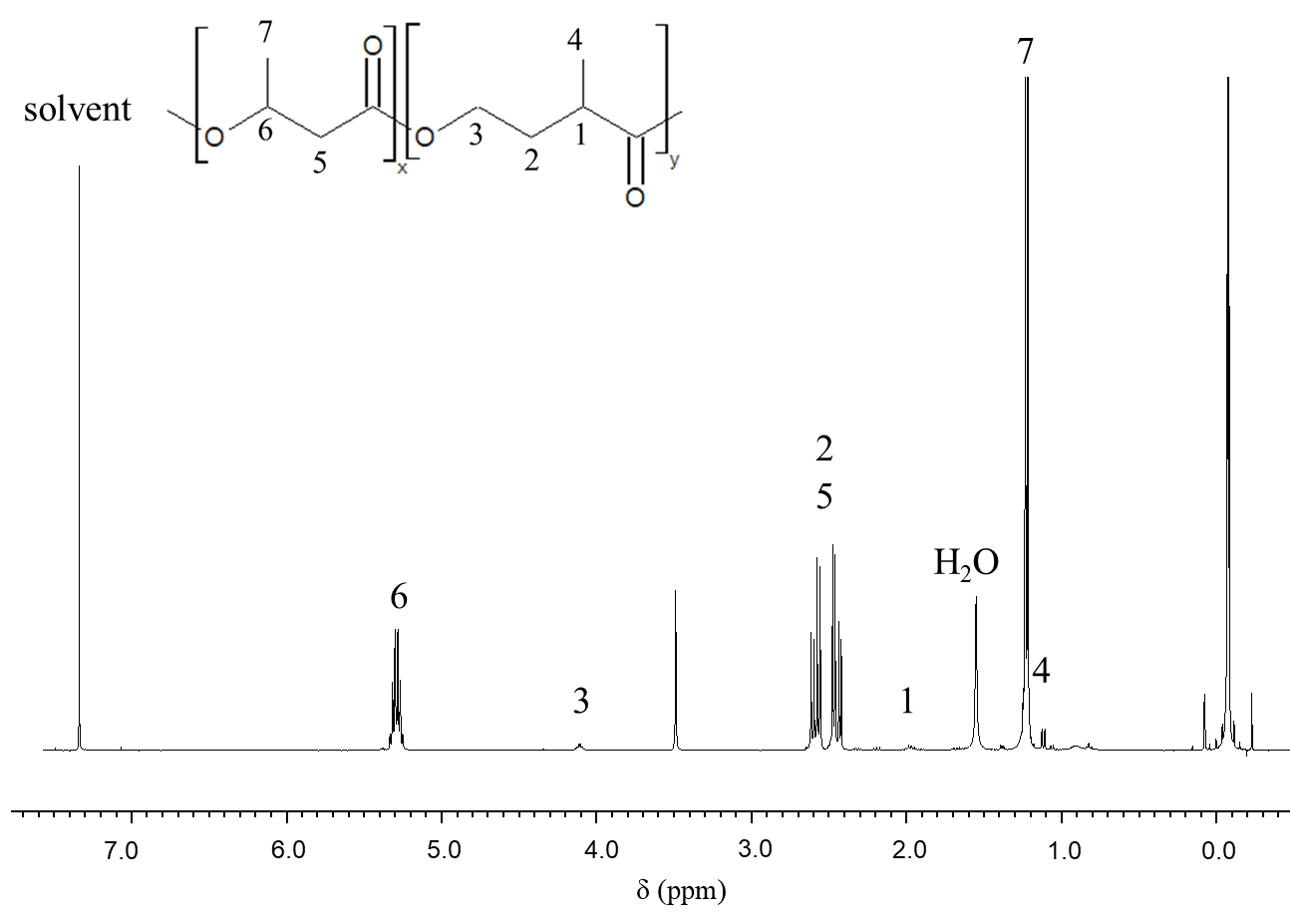


**Fig. S4D** ^１^H NMR of P(3HB-*co*-4H2MB)


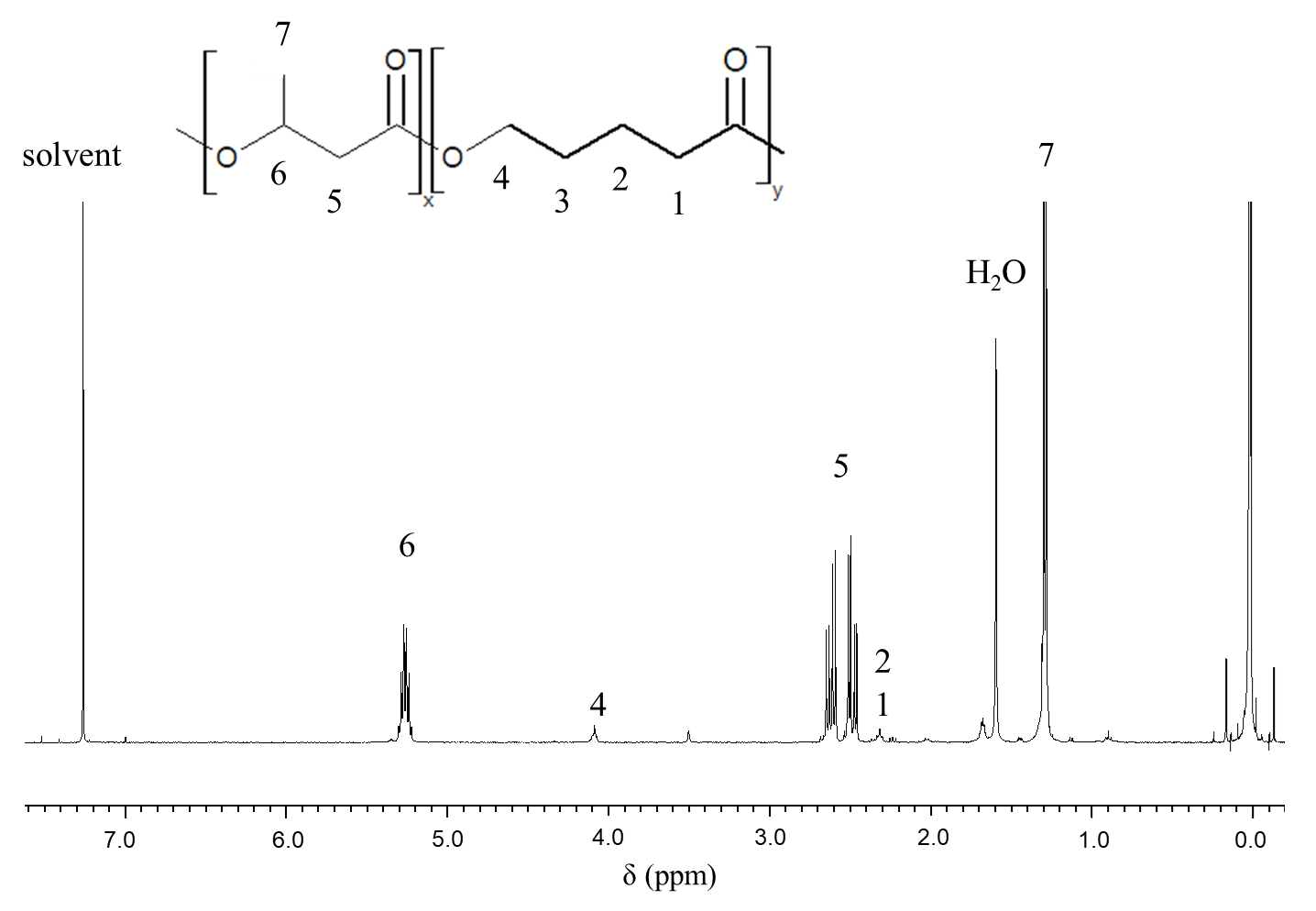


**Fig. S4E** ^１^H NMR of P(3HB-*co*­-5HV)


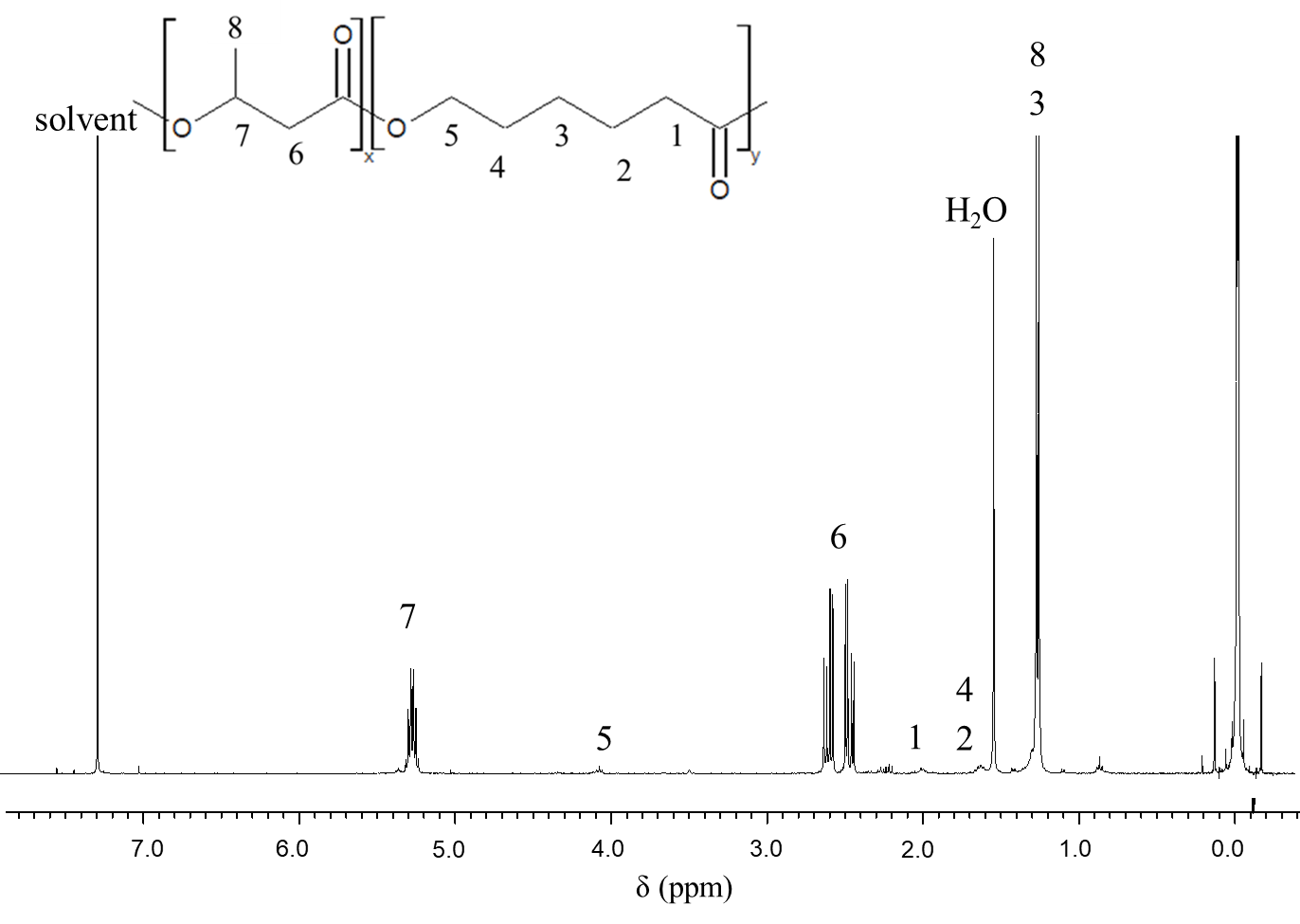


**Fig. S4F** ^１^H NMR of P(3HB-*co*-6HHx)


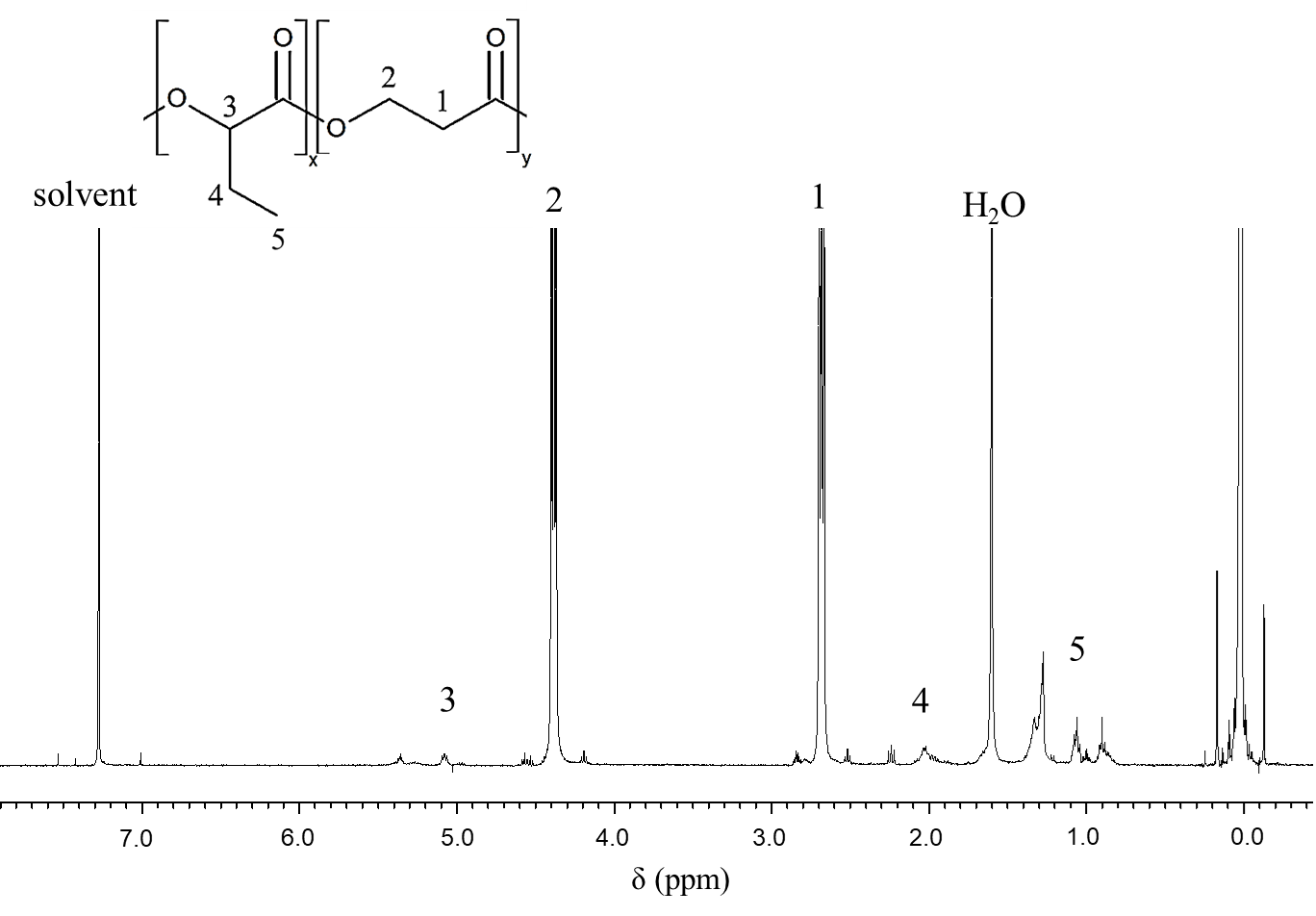


**Fig. S4G** ^１^H NMR of P(2HB-*co*-3HP)


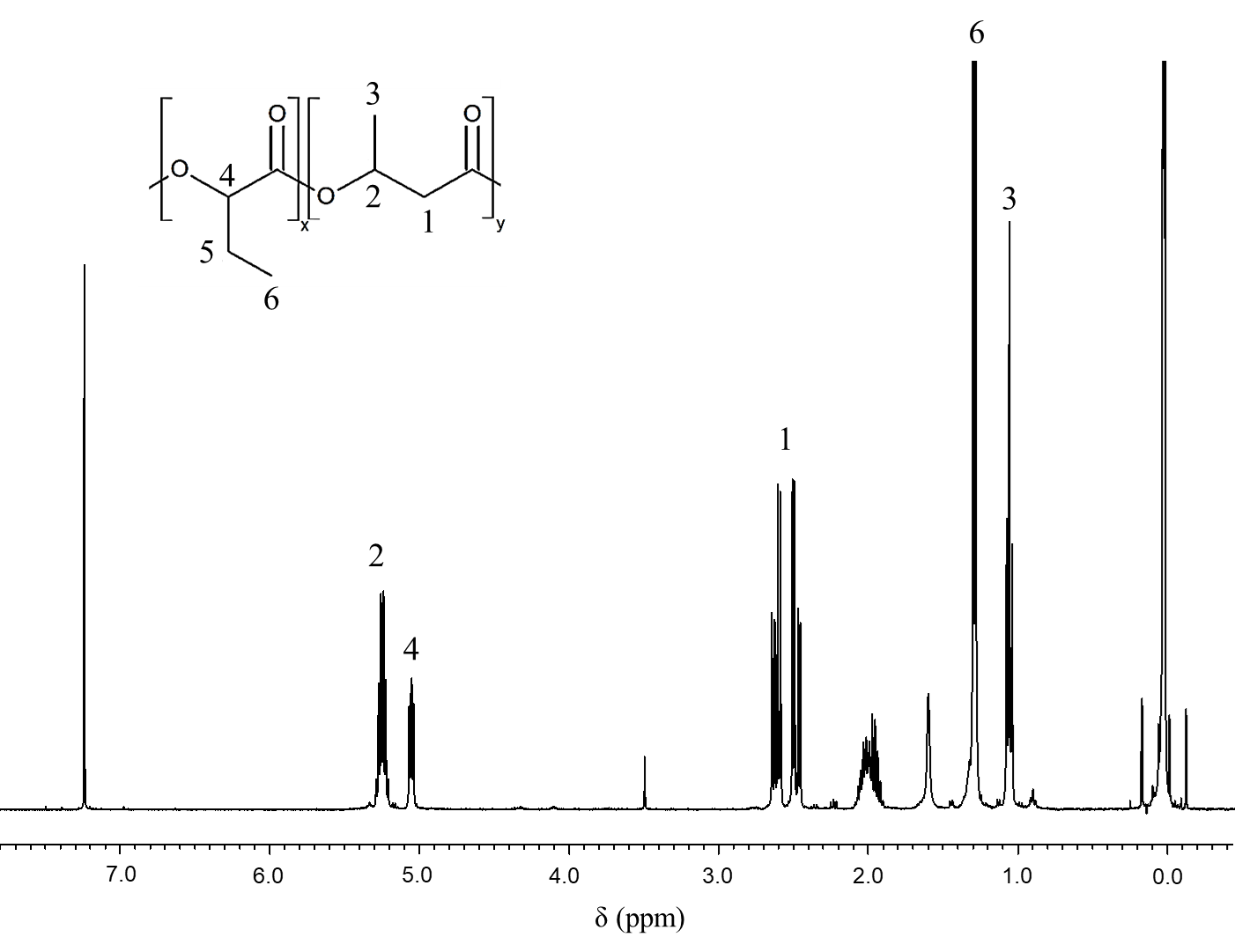


**Fig. S4H** ^１^H NMR of P(2HB-*co*-3HB)


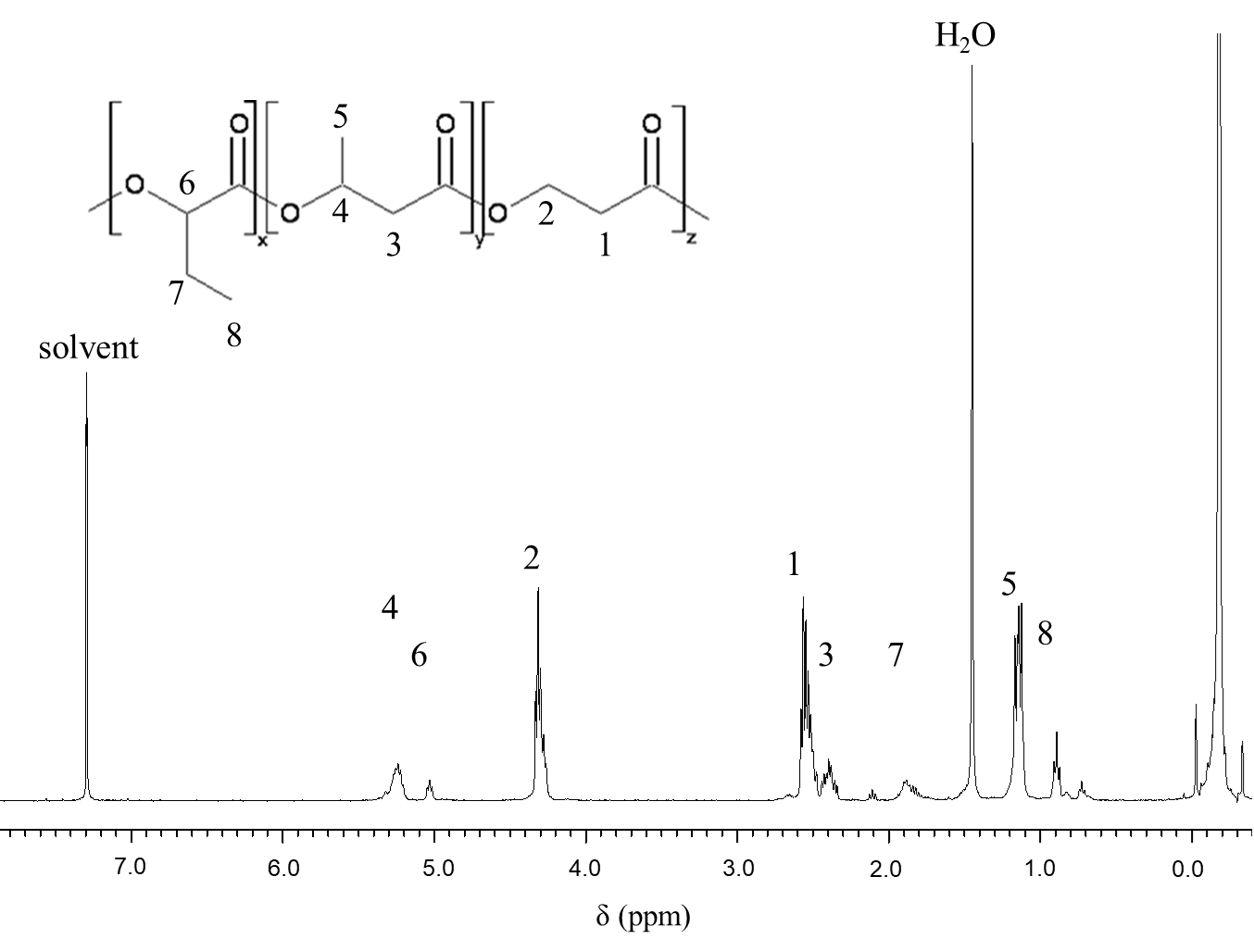


**Fig. S4I** ^１^H NMR results for P(2HB-*co*-3HB-*co*-3HP)


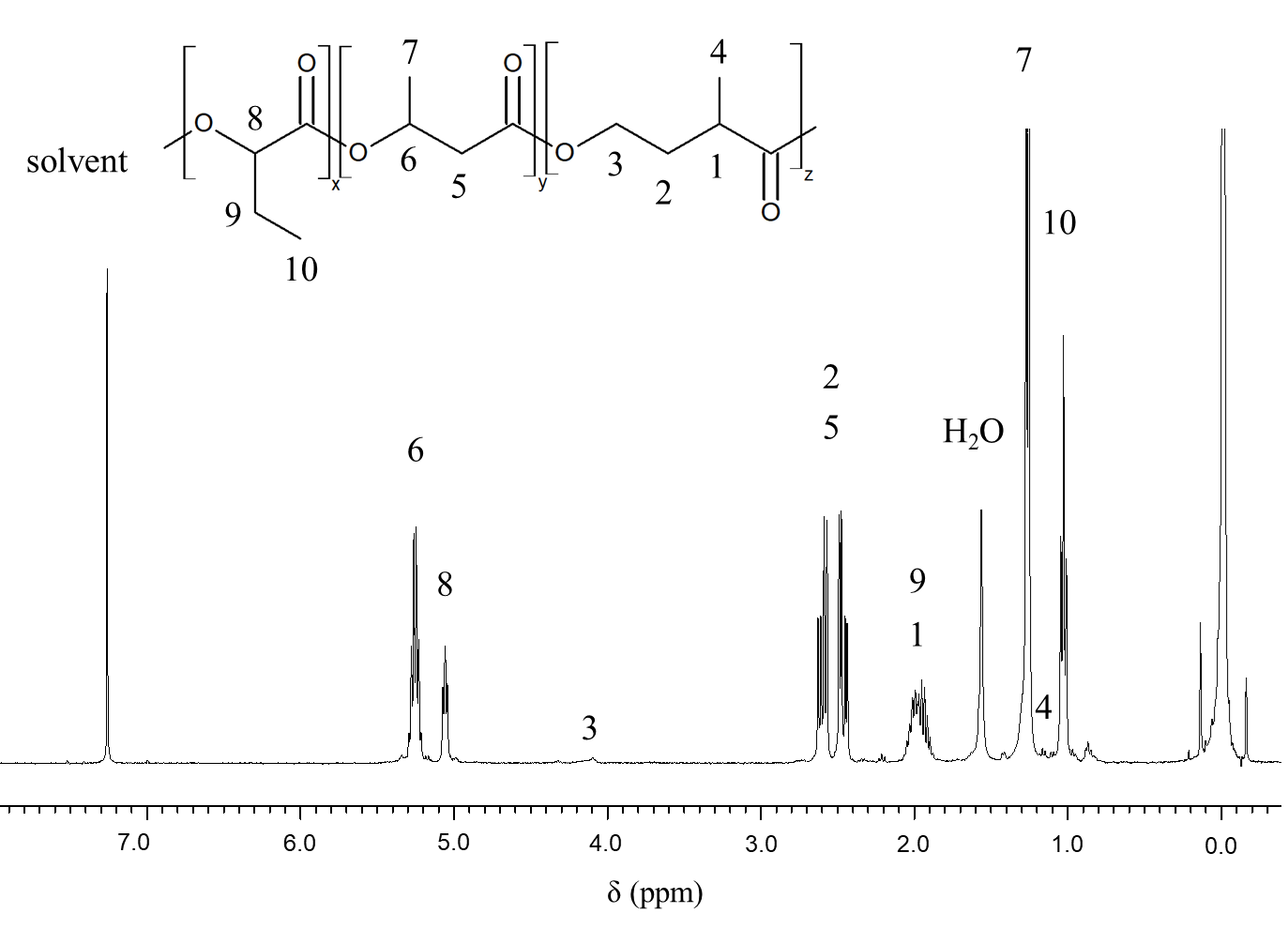


**Fig. S4J** ^１^H NMR of P(2HB-*co*-3HB-*co*-4H2MB)


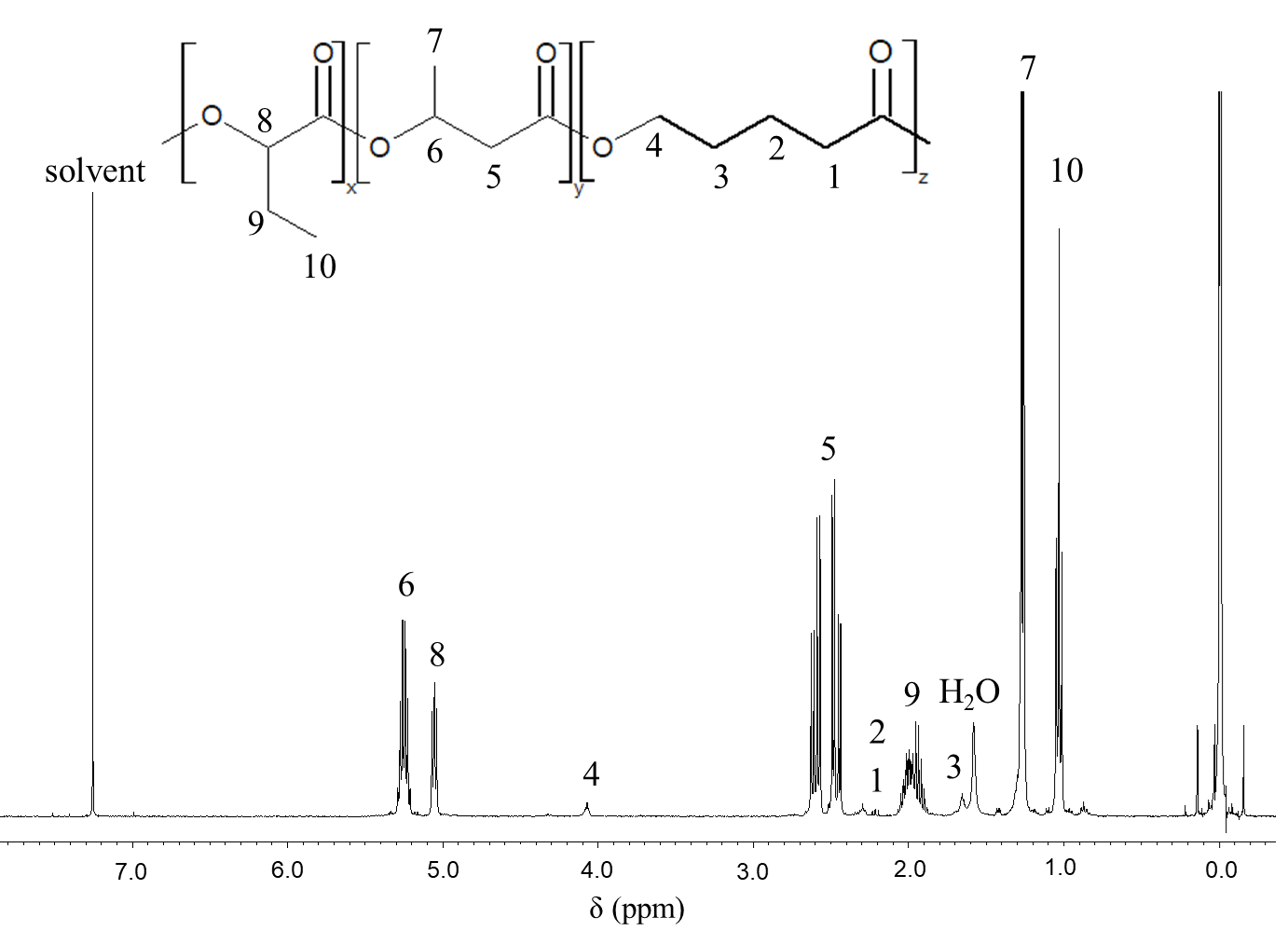


**Fig. S4K** ^１^H NMR of P(2HB-*co*-3HB-*co-*5HV)


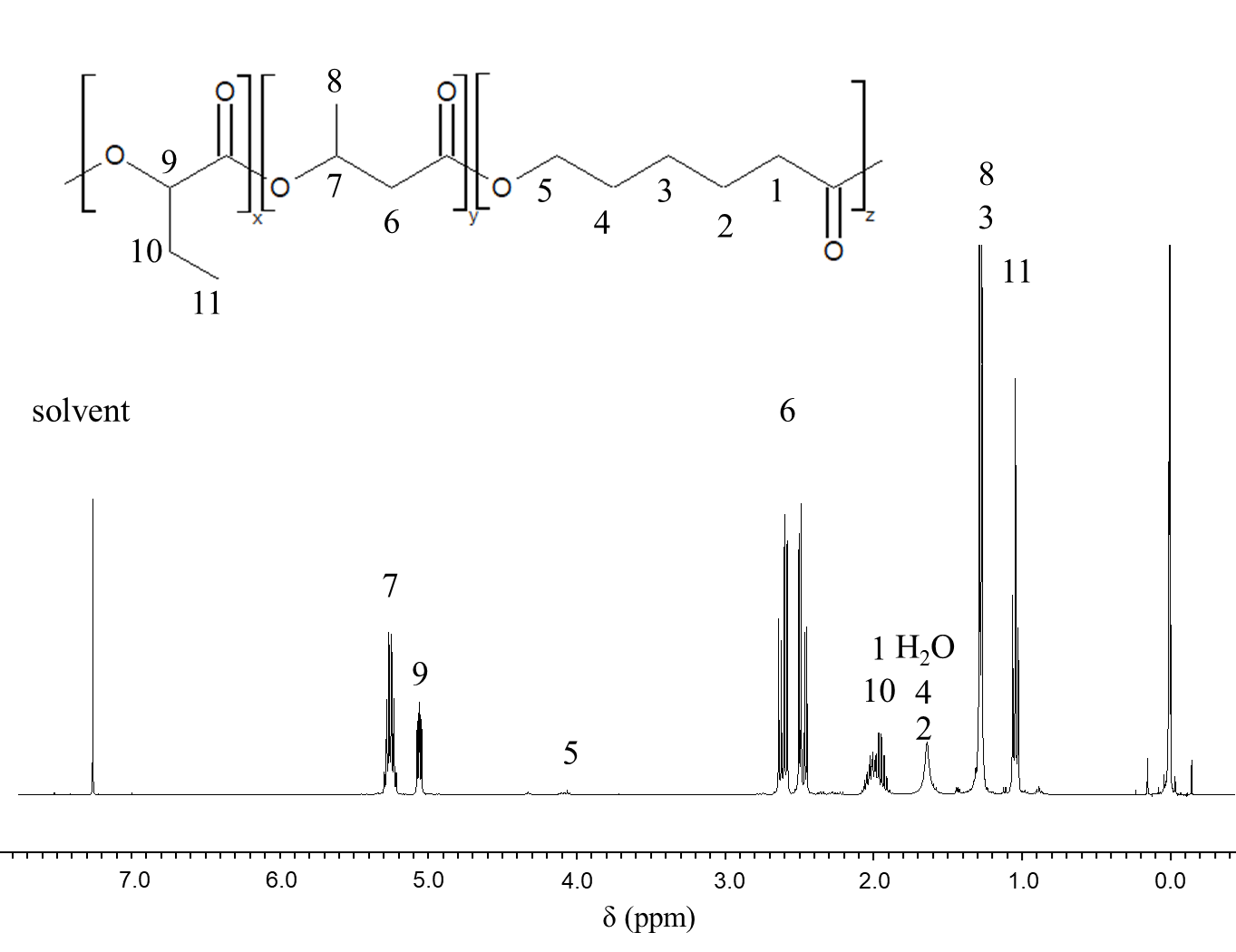


**Fig. S4L** ^１^H NMR of P(2HB-*co*-3HB-*co*-6HHx)


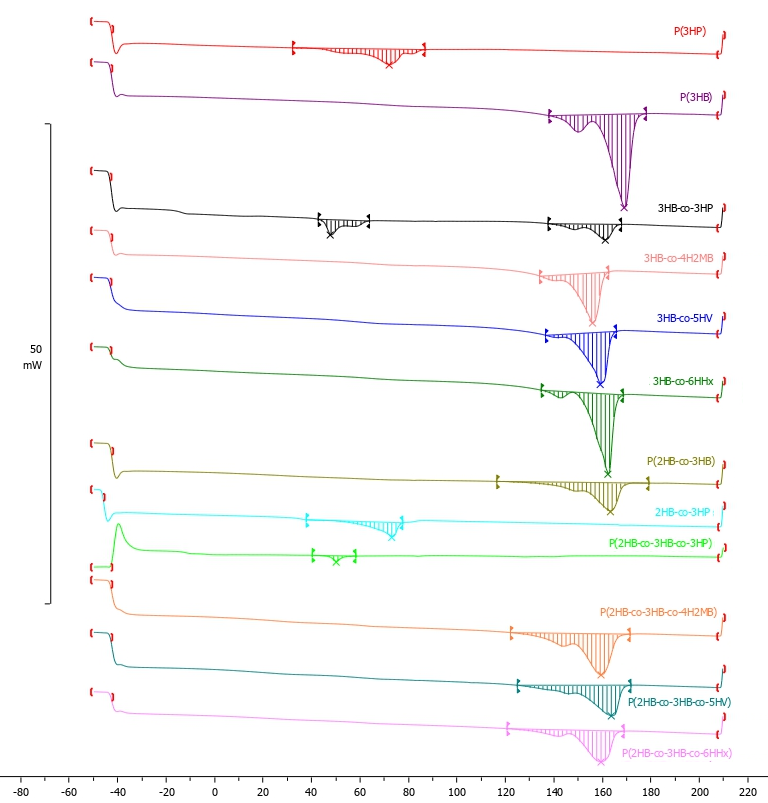


**Fig. S5A** DSC thermograms (1^st^ heating scan) of polymers in Table 3. The monomer compositions of the polymers are shown in Table 3.


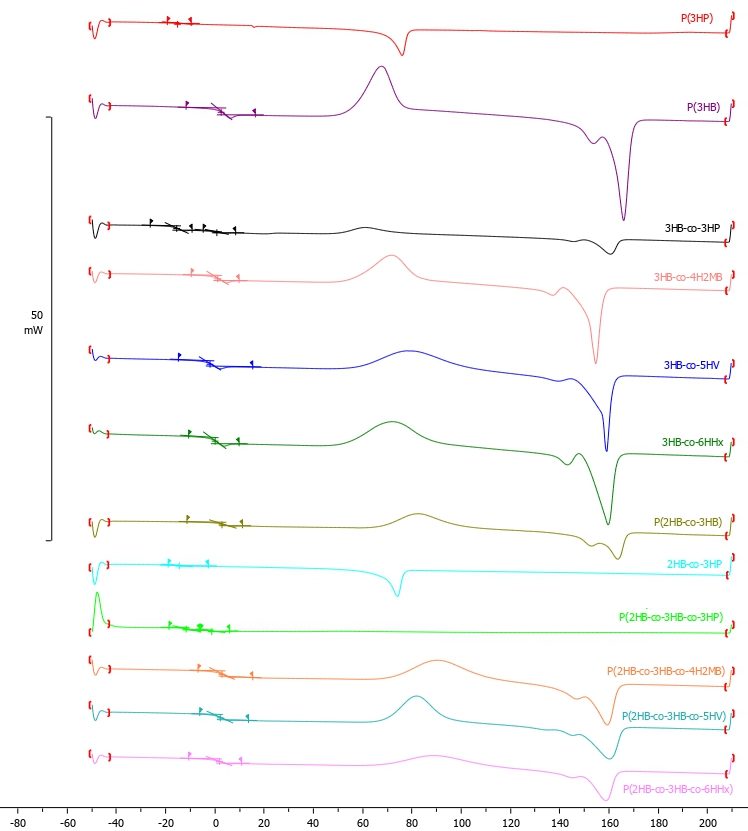


**Fig. S5B** DSC thermograms (2^nd^ heating scan) of polymers in Table 3. The monomer compositions of the polymers are shown in Table 3.
